# Enhanced upgrading of lignocellulosic substrates by coculture of *Saccharomyces cerevisiae* and *Acinetobacter baylyi* ADP1

**DOI:** 10.1186/s13068-024-02510-8

**Published:** 2024-05-06

**Authors:** Changshuo Liu, Bohyun Choi, Elena Efimova, Yvonne Nygård, Suvi Santala

**Affiliations:** 1 Faculty of Engineering and Natural Sciences, Tampere University, Hervanta Campus, Tampere, Finland; 2 Department of Life Sciences, Industrial Biotechnology, Chalmers University of Technology, Gothenburg, Sweden; 3 VTT Technical Research Centre of Finland, Espoo, Finland

**Keywords:** Lactic acid, Cocultivation, Detoxification, *Acinetobacter baylyi* ADP1, *Saccharomyces cerevisiae*, Wax esters, Lignocellulose

## Abstract

**Background:**

Lignocellulosic biomass as feedstock has a huge potential for biochemical production. Still, efficient utilization of hydrolysates derived from lignocellulose is challenged by their complex and heterogeneous composition and the presence of inhibitory compounds, such as furan aldehydes. Using microbial consortia where two specialized microbes complement each other could serve as a potential approach to improve the efficiency of lignocellulosic biomass upgrading.

**Results:**

This study describes the simultaneous inhibitor detoxification and production of lactic acid and wax esters from a synthetic lignocellulosic hydrolysate by a defined coculture of engineered *Saccharomyces cerevisiae* and *Acinetobacter baylyi* ADP1. *A. baylyi* ADP1 showed efficient bioconversion of furan aldehydes present in the hydrolysate, namely furfural and 5-hydroxymethylfurfural, and did not compete for substrates with *S. cerevisiae*, highlighting its potential as a coculture partner. Furthermore, the remaining carbon sources and byproducts of *S. cerevisiae* were directed to wax ester production by *A. baylyi* ADP1. The lactic acid productivity of *S. cerevisiae* was improved approximately 1.5-fold (to 0.41 ± 0.08 g/L/h) in the coculture with *A. baylyi* ADP1, compared to a monoculture of *S. cerevisiae*.

**Conclusion:**

The coculture of yeast and bacterium was shown to improve the consumption of lignocellulosic substrates and the productivity of lactic acid from a synthetic lignocellulosic hydrolysate. The high detoxification capacity and the ability to produce high-value products by *A. baylyi* ADP1 demonstrates the strain to be a potential candidate for coculture to increase production efficiency and economics of *S. cerevisiae* fermentations.

**Supplementary Information:**

The online version contains supplementary material available at 10.1186/s13068-024-02510-8.

## Background

Lignocellulosic biomass provides an abundant renewable resource and is expected to become increasingly important in the future society as a complementary and alternative to fossil-based feedstocks [1]. Nonetheless, lignocellulosic biomass is a challenging substrate for biorefinery applications as it is recalcitrant and thus needs pretreatment for depolymerizing hemicellulose and releasing the sugars [2]. The pretreatment of lignocellulosic biomass, e.g., by acidic thermochemical hydrolysis also generates various byproducts, including aliphatic acids, phenolic acids, and furan derivatives [3]. This results in a complex raw material which is a great challenge when lignocellulosic hydrolysates are converted to bioproducts through fermentation [3].

The complex composition of lignocellulosic hydrolysates challenges the comprehensive consumption of all available carbon sources, which can result in low overall carbon yields and poor economic feasibility. Moreover, the metabolism of some carbon sources can readily result in the generation of byproducts. *Saccharomyces cerevisiae* is a Crabtree positive yeast, e.g., it produces ethanol also during respiration. Similarly, acetic acid is a natural byproduct of ethanol fermentation by *S. cerevisiae*, but also an inhibitory compound even at low concentrations [4]. Metabolic engineering is a potential approach to broaden the substrate range and to prevent byproduct formation, but it can cause additional metabolic burden on the host cell, potentially causing decreased robustness and loss of function in harsh bioprocess conditions [5].

The inhibitors released during the pretreatment process, such as furan derivatives, aliphatic acids, and phenolic acids, are toxic to the microbial hosts and can negatively affect the cell performance in the process. For example, the toxicity of furan derivatives, such as furfural and 5-hydroxymethylfurfural (HMF), has been reported to inhibit central carbon metabolism of *S. cerevisiae* [6]. To overcome the toxicity, some microbes have evolved mechanisms enabling them to grow in the presence of inhibitors, through for instance detoxifying the inhibitors. However, the reactions typically require energy and resources that may hamper growth and production efficiency. For example, the growth of *Escherichia coli* upon furfural exposure was shown to be inhibited due to deficit of NAD(P)H [7, 8], as these energy carriers are consumed during furfural reduction. In addition to competition for NAD(P)H, rapid consumption of ATP was observed in the cultivation of *S. cerevisiae* in the presence of furfural and HMF [7, 9].

To allow more efficient and economically feasible use of lignocellulosic biomass as feedstock, new process innovations are needed. To that end, rationally designed microbial consortia could be a potential approach to overcome the challenges related to the mixed carbon sources and the presence of inhibitory compounds in lignocellulose hydrolysates [10, 11]. Brethauer et al. demonstrated a three-strain consortium for the simultaneous production of sugars and ethanol from a pretreated wheat straw using a membrane biofilm reactor, in which *Trichoderma reesei* first produced glucose and xylose from which *S. cerevisiae* and *Scheffersomyces stipites* produced ethanol, respectively [12]. In another example, a cocultivation of ethanologenic, xylose-consuming *E. coli* unable to utilize glucose, and *S. cerevisiae* was reported to efficiently produce ethanol from sugar cane bagasse [13]. To demonstrate the improved and simultaneous detoxification and bioproduction, a microbial consortium was established using xylose-utilizing and inhibitor-tolerant *S. cerevisiae* strains, resulting in simultaneous detoxication of furan derivatives and increased ethanol production [14].

*Saccharomyces cerevisiae*, being the most well-known model yeast, has been used for the production of a wide range of commodities from lignocellulosic hydrolysates, including lactic acid [15]. Lactic acid has been used in diverse applications such as food, pharmaceuticals, textiles, and chemical industries [16]. Notably, lactic acid is polymerized to polylactic acid (PLA), a biodegradable and biocompatible bioplastic [17]. Lactic acid can be produced through chemical or microbial fermentative processes. Today, over 90% of the lactic acid is produced through microbial fermentation of sugars derived from sugarcane or corn starch, thus competing for resources with food and feed applications [18]. Therefore, agricultural and forest residues, e.g., lignocellulosic biomass, would be an attractive alternative as a sustainable and renewable feedstock for lactic acid production.

The strictly aerobic soil bacterium *Acinetobacter baylyi* ADP1 (later ADP1) possesses a substrate spectrum complementary to that of *S. cerevisiae*. ADP1 can natively utilize carbon sources in the lignocellulosic hydrolysates such as phenolic acids [19, 20] that *S. cerevisiae* cannot use. In addition, acetate and ethanol, that are typical byproducts of *S. cerevisiae*, are preferred carbon sources of ADP1. Conversely, ADP1 cannot utilize sugars other than glucose because it does not possess a complete Embden–Meyerhof–Parnas glycolytic pathway [21]. Moreover, biotransformation of furfural and HMF to less toxic compounds by *Acinetobacter* strains has been reported [22–24]. In addition to the wide substrate spectrum, ADP1 can produce interesting high-value products, such as wax esters (WEs) [25], used in broad range of applications including cosmetics, lubricants, and pharmaceuticals. The production of WEs has been previously demonstrated from, e.g., acetate [26–28] and phenolic compounds [29]. In addition, ADP1 can be readily engineered due to its natural competence and the wide range of available genetic tools [30]. Considering the metabolic features of ADP1, it could serve as a potential fermentation companion for *S. cerevisiae*. ADP1 has been previously successfully employed in a number of different cocultures, for example as the detoxifier for a lignocellulosic hydrolysate [31, 32] and to improve growth, production, and carbon recovery [33–36].

In this study, we developed a coculture of ADP1 and *S. cerevisiae* for the production of lactic acid from a synthetic lignocellulose hydrolysate. In the consortium, *S. cerevisiae* efficiently utilized the sugars and produced lactic acid whereas ADP1 consumed the residual carbon of substrates and the byproducts from *S. cerevisiae*. In addition to the lactic acid produced by *S. cerevisiae*, ADP1 directed the residual carbon to high-value lipids, namely WEs. By the developed approach, we demonstrate the potential of employing cocultures for improved production and carbon utilization in complex and challenging feedstock, such as lignocellulose hydrolysates.

## Methods

### Strains

A wild-type *Acinetobacter baylyi* ADP1 (DSM 24193, DSMZ, Germany), a transposon-free *A. baylyi* ADP1-ISx (a kind gift from the Barrick lab) [37], *Saccharomyces cerevisiae* CEN. PK XXX [38], and strains derived from these were used in this study (Table 1).

**Table 1:** Strains used in this study

Strain designation	Description	References
*Acinetobacter baylyi* ADP1		
ADP1	Wild-type *A. baylyi* ADP1	DSM 24193, DSMZ
ADP1-ISx	Transposon-free *A. baylyi* ADP1	[37]
ASA507	*A. baylyi* ADP1-ISx, hcaE*, hcaK*. The parental strain of ASA707 and ASA714. Highly ferulate tolerant	[39]
ASA711	ASA507; Δ*lldPRD, dld;* Δ*﻿acr1::P*_*t5*_*-acr1-kan*^*R*^	This study
ASA714	ASA711; Δ*poxB::mScarlet-cm*^*R*^	This study
*Saccharomyces cerevisiae*		
CEN. PK XXX	A diploid, xylose-fermenting strain based on *S. cerevisiae* CEN. PK 122 MDS	[38]
CEN. PK LX1	CEN. PK XXX, Δ*cyb2::pTDH3-ldh-tCYC1*	This study
CEN. PK LX2	CEN. PK LX1, Δ*erf2::pTDH3-ldh-tCYC1*	This study
CEN. PK LX3	CEN. PK LX2, Δ*gpd1::pTEF2-ldh-tCYC1*	This study

### Media and culture conditions

#### Preparation of the synthetic lignocellulosic hydrolysate

The synthetic lignocellulosic hydrolysate (SLH) was designed to mimic the composition of spruce hydrolysate (described by Nickel [40]), when diluted to 70%. The composition of the SLH medium was the following: d-glucose 12.18 g/L, d-xylose 7.91 g/L, d-mannose 10.43 g/L, d-galactose 7.14 g/L, acetic acid 2.66 g/L, ferulic acid 0.5 g/L, *p*-coumaric acid 0.5 g/L, furfural 0.84 g/L, HMF 0.35 g/L, formic acid 0.28 g/L, yeast extract 5 g/L, urea 2.5 g/L, (NH_4_)_2_SO_4_ 2.5 g/L. A modified SLH medium was used for pre-cultivations, e.g., SLH medium with the half amount of furfural and HMF (0.42 g/L and 0.175 g/L, respectively). The pH of the SLH medium was adjusted to 7.0 using 5 M NaOH.

#### Media and culture conditions for ADP1

ADP1 cells were used for cloning and transformation purposes grown in 14 mL culture tubes with 5 mL modified low salt Lysogeny broth (LB) medium (tryptone 10 g/L, yeast extract 5 g/L, NaCl 1 g/L), or LB-agar plates (adding 15 g/L agar to the modified low salt LB medium) supplemented with 1% (*w/v*) glucose and corresponding antibiotics (chloramphenicol 25 µg/mL, kanamycin 30 µg/mL). The liquid cultivations were incubated at 30 °C and 300 rpm in an Innova 44 shaker (Eppendorf, Germany), cultivations using LB-agar plates were incubated at 30 °C. For the *tdk/kan*^*R*^ rescue screening purpose, the solid medium was supplemented with 400 µg/mL azidothymidine.

For strain characterization and determining the biotransformation of furan aldehydes by ADP1, all pre-cultures were inoculated from LB-agar plates, grown overnight in 10 mL modified SLH medium in 100 mL shake flasks at 30 °C and 300 rpm in an Innova 44 shaker (Eppendorf, Germany). Cells collected from the overnight pre-cultures were used to inoculate 50 mL fresh SLH medium in 250 mL shake flasks with an initial optical density at 600 nm (OD_600_) of 1.0 and incubated at 30 °C and 300 rpm. Samples of 1 mL were collected every hour by centrifugation at 13,500×*g* for 3 min, after which the supernatants were analyzed by high-performance liquid chromatography (HPLC).

#### Media and culture conditions for *S. cerevisiae*

*Saccharomyces cerevisiae* cells were cultured for cloning and transformation purposes in 14 mL culture tubes with 3 mL yeast peptone (YP) medium (yeast extract 10 g/L, peptone 20 g/L) with varying carbon sources. The YPD medium contained glucose at 20 or 50 g/L, YPX xylose at 20 or 50 g/L, and YPDX glucose and xylose at 10 g/L each. Solid media for *S. cerevisiae* was prepared by adding 20 g/L agar and 200 µg/L Geneticin (Fisher Scientific, MA, USA) to the YPD medium. All *S. cerevisiae* cultures were incubated at 30 °C, liquid cultivations were shaken at 200 rpm in an Innova 44 shaker (Eppendorf, Germany).

All *S. cerevisiae* pre-cultures for strain characterization were inoculated from solid medium cultures, grown overnight (~ 16 h) in 3 mL YPD (20 g/L glucose), YPX (20 g/L xylose), YPDX (10 g/L glucose and 10 g/L xylose), or SLH medium in 14 mL culture tubes. The cells were collected, washed with sterilized water, and used for inoculation of liquid cultures in YPD, YPX, YPDX, or SLH medium. The flask scale liquid cultures were grown in 40 mL medium in 250 mL Erlenmeyer flasks with an initial OD_600_ of 0.1. The 250 µL microtiter plate cultures were grown in 96-well plates, with an initial OD_600_ of 0.1 at 30 °C and 250 rpm in a growth profiler 960 device (Enzyscreen, Netherlands). Various concentrations (0 to 1000 mM) of l-(+)-lactic acid (Sigma Aldrich, USA) solutions were added to the YPD media for examination of lactic acid tolerance. The initial pH of the YPD medium supplemented with lactic acid was adjusted to 6.0 with 2 M NaOH solution. Varying amount of furfural (0 to 1.5 g/L), HMF (0 to 1 g/L), or mixture of furfural and HMF (0 to 1 g/L of each) were used for evaluation of tolerance to inhibitors. For the assays for measuring biotransformation of furan-derived compounds, the same condition was applied to LX3 as to ADP1.

#### Bioreactors

Cells of LX3 and ASA714 were inoculated from solid YPD and LB-agar plate, respectively. Two subsequent overnight pre-cultures were used to prepare cells for the cocultivation. The first pre-cultivations were performed in 25 mL modified SLH medium in 200 mL shake flasks, followed by a second pre-cultivation with an initial OD_600_ of 1, in 50 mL SLH medium in 500 mL shake flasks. The pre-cultures were incubated at 30 °C and 200 rpm for 20 h. Baffled Erlenmeyer flasks were used for ADP1 cultures, whereas shake flasks were used for the *S. cerevisiae* cultures. The cocultivations were carried out in 3.5-L bioreactors (Biostream International BV, Netherlands) with SLH medium supplemented with 1 mL antifoaming agent (Antifoam 204, Sigma Aldrich). The working volume of the bioreactor was 1 L, and the cultivations were carried out at 30 °C. The minimal dissolved oxygen level was set to 30%, controlled by automatically adjusting the agitation from 300 to 900 rpm. The culture pH was maintained at 7.0 using 2 M HCl and 8% (v/v) NH_4_OH. Control fermentations using monoculture of LX3 with same experimental conditions were also conducted. Samples were collected at different times between 0 h (inoculation) to 56 h (end of fermentation). All bioreactor cultivations were repeated four times.

### Strain construction

#### Engineering ADP1

The transformation and homologous recombination-based genome editing of ADP1 were done as described by Santala et al. [41]. The parental strain ASA507 was constructed and described in our previous study by Luo et al. [39]. To construct a markerless, lactate utilization negative ADP1 strain, a counter selection method based on the *tdk/kan*^*R*^ cassette [42] was applied. The lactate dehydrogenase operon (genes *lldPRD, dld*; ACIAD 0106-0109) knockout cassette with *tdk/kan*^*R*^ and the rescue cassette were constructed using splicing by overlap-extension PCR. Briefly, approximately 1000 bp of homologous sequences upstream and downstream of the lactate dehydrogenase operon were directly amplified from genomic DNA of wild-type ADP1, with approximately 20 bp overlapping sequences homologous to the *tdk/kan*^*R*^ cassette. The *tdk/kan*^*R*^ cassette was amplified from the genome of ADP1 Δ*acr1*::*tdk/kan*^*R*^ [43] (a kind gift from Veronique de Berardinis, Genoscope, France). Counter selection of the markerless strain was carried out as described previously [42]. The obtained strain, ASA707, was used as the parental strain for overexpression of the wax ester production pathway. First, a knockout cassette of *acr1* was amplified from ADP1 Δ*acr1*::*tdk/kan*^*R*^ as described previously [44] and used for transformation of ASA707 to obtain ASA710. For the overexpression of *acr1,* an integration cassette *P*_*t5*_*-acr1-kan*^*R*^ was amplified from the genome of a previously described strain ASA523 [45] and used to transform ASA710 to obtain ASA711. Next, the gene encoding the mScarlet fluorescence protein was integrated to ASA711 at the *poxB* (ACIAD 3381) site using a gene cassette described previously [36] to obtain the strain ASA714 used in this study. The genotype of all the constructions was verified using PCR and phenotype tests. All oligonucleotides were ordered from Thermo Scientific (USA). All the primers and plasmids used in this study are listed in Additional file 1: Tables S1 and S2, respectively. The PCR reagents were provided by Thermo Scientific (USA).

#### Engineering *S. cerevisiae*

The lactic-acid-producing *S. cerevisiae* strain was derived from the xylose-fermenting *S. cerevisiae* CEN. PK XXX strain [38]. The *LDH* gene from *Bos taurus* was synthesized with codon optimization for *S. cerevisiae* (by IDT, USA)*.* The LDH expression module was constructed in the yIplac128 and yIplac204 plasmids [46] which contained *LDH* under control of the *TDH3* and *TEF2* promoters and the *CYC1* terminator, resulting in plasmids YIP_BC01 (yIplac128-*TDH3p-LDH-CYC1t*) and YIP_BC02 (yIplac204-*TEF2p-LDH-CYC1t*), respectively*.* All genetic modifications were introduced using CRISPR/Cas9-mediated genome editing as previously described [47]. In brief, replacement of *GPD1*, *ERF2*, or *CYB2* was achieved by integration of PCR amplified expression cassettes from yIplac128-*TDH3p-LDH-CYC1t* or yIplac204-*TEF2p-LDH-CYC1t* using primers containing 40 bp homologous sequences flanking the targeted genes. The *LDH* expression cassette *TDH3p-LDH-CYCt1* was integrated at the deletion site of *CYB2* and *GPD1*, and *TEF2p-LDH-CYC1t* was inserted by replacement of *ERF2*. The resulting strain was named LX3 and had six copies of *LDH* integrated in the genome. The purified PCR products were co-transformed [48] with the YN2_1 plasmid [47] expressing Cas9 and sgRNAs targeting *GPD1, ERF2, or CYB2.* The protospacer sequences were chosen using CRISPR-ERA [49]. All strains were confirmed by PCR, oligonucleotide synthesis and Sanger sequencing of PCR products and plasmids were done by Eurofins genomics (by Eurofins, Luxembourg). All the primers and plasmids used in this study are listed in Additional file 1: Tables S1 and S2, respectively. The PCR reagents were provided by Thermo Scientific (USA).

### Analytical methods

Filtered (0.2 µm filers by Agilent Technologies, USA) culture supernatants were analyzed using HPLC. Furfural and HMF of the samples from the biotransformation assays were detected with an HPLC device (LC-40D Shimadzu, Japan) equipped with a Luna 5 µm C18 150 × 4.6 mm column (Phenomenex, USA), photo diode array detector (PDA*,* SPD-M40) and using water:methanol:formic acid solution (80: 20: 0.16, *v/v/v*) as mobile phase. The flow rate was 1.0 mL/min, and the column was maintained at 40 °C. Ferulic acid, coumaric acid, furfural, and HMF of the samples from the bioreactors were detected with a Dionex VWD-3400RS variable wavelength detector and Rezex RFQ-Fast Acid H^+^ (8%) Ion Exclusion column (7.8 × 100 mm, Phenomenex, USA). The column temperature was maintained at 80 °C and the flow rate was 0.6 mL/min, with 5 mM H_2_SO_4_ as an isocratic mobile phase. Ferulic acid and coumaric acid were detected at 254 nm, while furfural and HMF were detected at 280 nm. The concentration of lactic acid, xylose, glucose, acetic acid, and ethanol of the samples from the bioreactors was determined using a Dionex UltiMate 3000 series HPLC (ThermoFisher Scientific, USA) equipped with a Dionex RI-101 refractive index detector and an Aminex HPX-87H column (7.8 × 300 mm, Bio-Rad, USA) operating at 50 °C and 0.7 mL/min of a flow rate with 5 mM H_2_SO_4_ as an isocratic mobile phase.

WEs were quantified using ^1^H nuclear magnetic resonance (NMR) as described by Santala et al. [50]. An average molar mass of WEs of 506 g/mol was applied to WEs titer calculations [51].

## Results

We designed a defined coculture of *S. cerevisiae* and ADP1 for improved production of lactic acid from a synthetic lignocellulose hydrolysate. We hypothesized that using two metabolically diverse strains complementing each other, more efficient detoxification and comprehensive carbon utilization could be achieved. In addition, ADP1 could potentially, not only detoxify the furan-derived inhibitors, but also direct the carbon from aliphatic and phenolic acids and potential byproducts of *S. cerevisiae* to WE production, thus improving the overall carbon recovery and process feasibility. Importantly, while lactic acid is an extracellular product, WEs are accumulated intracellularly, therefore not interfering with the downstream process. Here, to establish the microbial consortium of *S. cerevisiae* and ADP1, we first constructed and characterized a lactic acid production strain *S. cerevisiae* CEN.PK LX3 and an engineered ADP1 strain ASA714. Thereafter, cocultivations were carried out in a bioreactor using SLH as the feedstock.

### Construction and characterization of ADP1

The tolerance of wild-type ADP1 on different concentrations of furfural or HMF was determined. Growth, albeit increasingly inhibited with increasing amounts of inhibitors, was observed in all culture conditions (Additional file 1: Fig. S1). The wild-type ADP1 was also able to grow in SLH medium (Additional file 1: Fig. S2). Expectedly, the wild-type ADP1 was capable of utilizing lactic acid, the target product of the designed process, as the sole carbon source (Additional file 1: Fig. S3).

To ensure ADP1 suitability and applicability for the coculture, genetic engineering was carried out. We used a previously constructed and characterized ADP1 strain, ASA507 [39], as the starting point. The strain exhibits improved tolerance and utilization of *p*-coumarate and ferulate, key aromatic monomers present in lignocellulose hydrolysates. We hypothesized that the high tolerance could positively affect the strain performance in SLH medium. First, the operon responsible for lactate utilization (genes *lldPRD*, *dld*; ACIAD 0106-0109) was deleted to prevent the utilization of the lactic acid produced by *S. cerevisiae*. In addition, to enable WE production in the coculture, we overexpressed *acr1* encoding for the key enzyme of the synthesis pathway, fatty acyl-CoA reductase Acr1, as we have previously shown it to improve the direction of carbon toward WE production [44]. Moreover, a gene encoding for the red fluorescent protein mScarlet was introduced to facilitate monitoring the strain growth and performance. The engineered strain was designated as ASA714. We confirmed that ASA714 did not grow on lactate as the sole carbon source, nor did it consume lactate in the presence of other carbon sources (such as acetate or other substrates of SLH medium; data not shown). Furthermore, the wax ester production of ASA714 was found to be higher compared to the wild-type ADP1 (Additional file 1: Fig. S4).

**Fig. 1:**
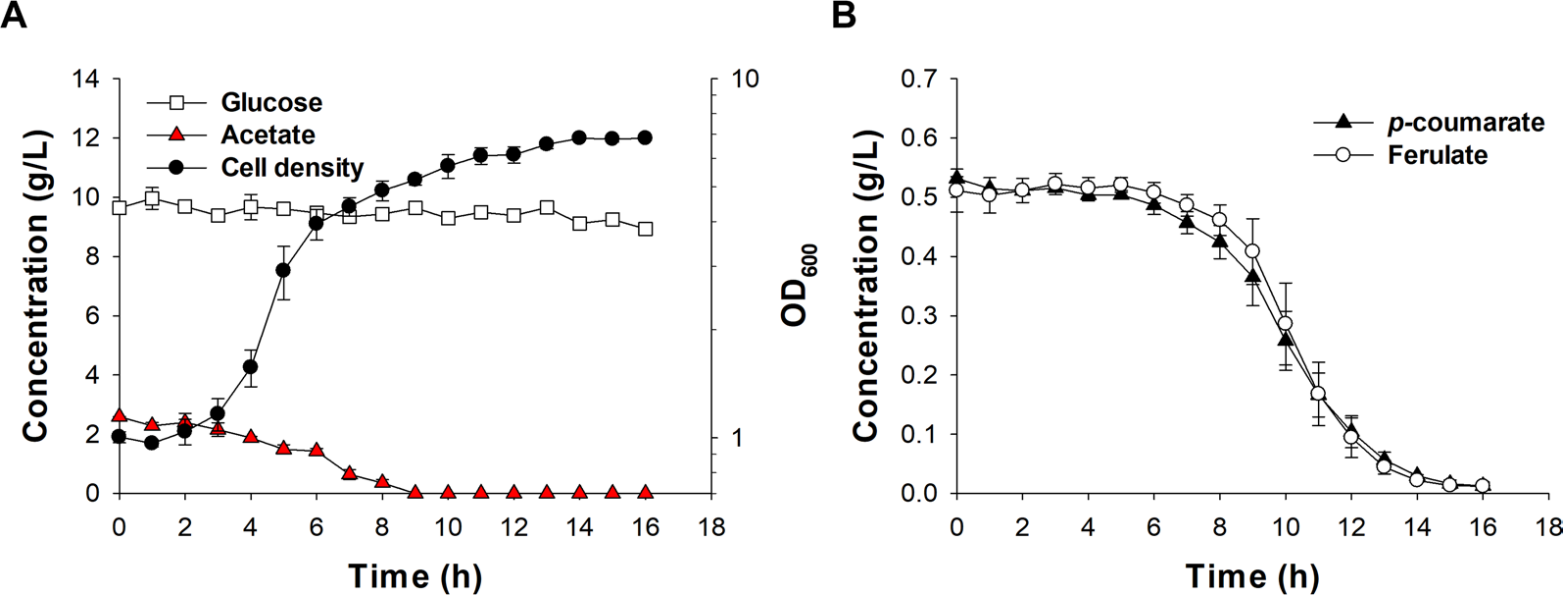
**A** Growth and consumption of glucose and acetate and **B** *p*-coumarate and ferulate of ASA714 in SLH medium. The experiment was repeated using independent biological replicates. The averages of the measurements, with error bars representing standard deviations are shown

We next determined the carbon utilization and growth of ASA714 in SLH medium (Fig. 1). A two-phased growth pattern was observed, where the maximum specific growth rate (*µ*_max_) of the first exponential phase (*µ*_max_ = 0.46 h^−1^, at 4–6 h) was much higher than the growth rate of the second phase (*µ*_max_ = 0.09 h^−1^, 6–11 h). The acetate of the SLH medium was completely consumed after 9 h, after which the aromatic compounds were consumed (Fig. 1). The consumption of glucose was negligible. Next, the lactic acid tolerance of ASA714 in SLH medium supplemented with different concentrations of lactate was studied (Additional file 1: Fig. S5). The growth rate of ASA714 was only slightly faster (*µ*_max_ = 0.18 h^−1^) in medium without lactate compared to medium supplemented with 18 g/L lactate (*µ*_max_ = 0.15 h^−1^), which was the highest concentration tested.

### Construction and evaluation of the lactic-acid-producing *S. cerevisiae* CEN.PK LX3

To obtain a *S. cerevisiae* strain with high lactic acid production from mixed monosaccharides, the xylose-fermenting strain CEN PK. XXX [38] was used as the parental strain. Prior to proceeding in strain engineering, XXX strain was evaluated as host strain for lactic acid production by characterization with mixed carbon supplementation, at various pHs, and increasing lactic acid concentrations. In fermentation at micro-scale, the growth of CEN PK. XXX in glucose, xylose, and a mixture of glucose and xylose was measured. The *µ*_max_ was 0.41 h^−1^ on glucose, 0.43 h^−1^ on xylose, and 0.43 h^−1^ on glucose and xylose (Additional file 1: Fig. S6A). During fermentation with glucose and a mixture of glucose and xylose, a diauxic growth pattern was observed. In medium with only xylose, a single exponential phase was observed. After 9 h of fermentation, 8.3 g/L and 4.5 g/L of ethanol were detected in media with glucose or glucose and xylose, respectively. In medium with only xylose, 1.4 g/L of ethanol was observed (Additional file 1: Fig. S6B). Next, the lactic acid tolerance of the XXX strain was examined, and no growth inhibition was observed at up to 250 mM (22.5 g/L) of lactic acid for 72 h fermentation (Additional file 1: Fig. S7). Nonetheless, lactic acid concentration over 250 mM presented inhibitory effect for cell growth with 0.19 h^−1^ (500 mM), and 0.16 h^−1^ (750 mM) of *µ*_max_, which represented 68% and 57% of the growth rates at 0 mM lactic acid (0.29 h^−1^). The highest concentration of lactic acid (1000 mM, 90 g/L) showed a 70% growth inhibition (Additional file 1: Fig. S7).

**Fig. 2:**
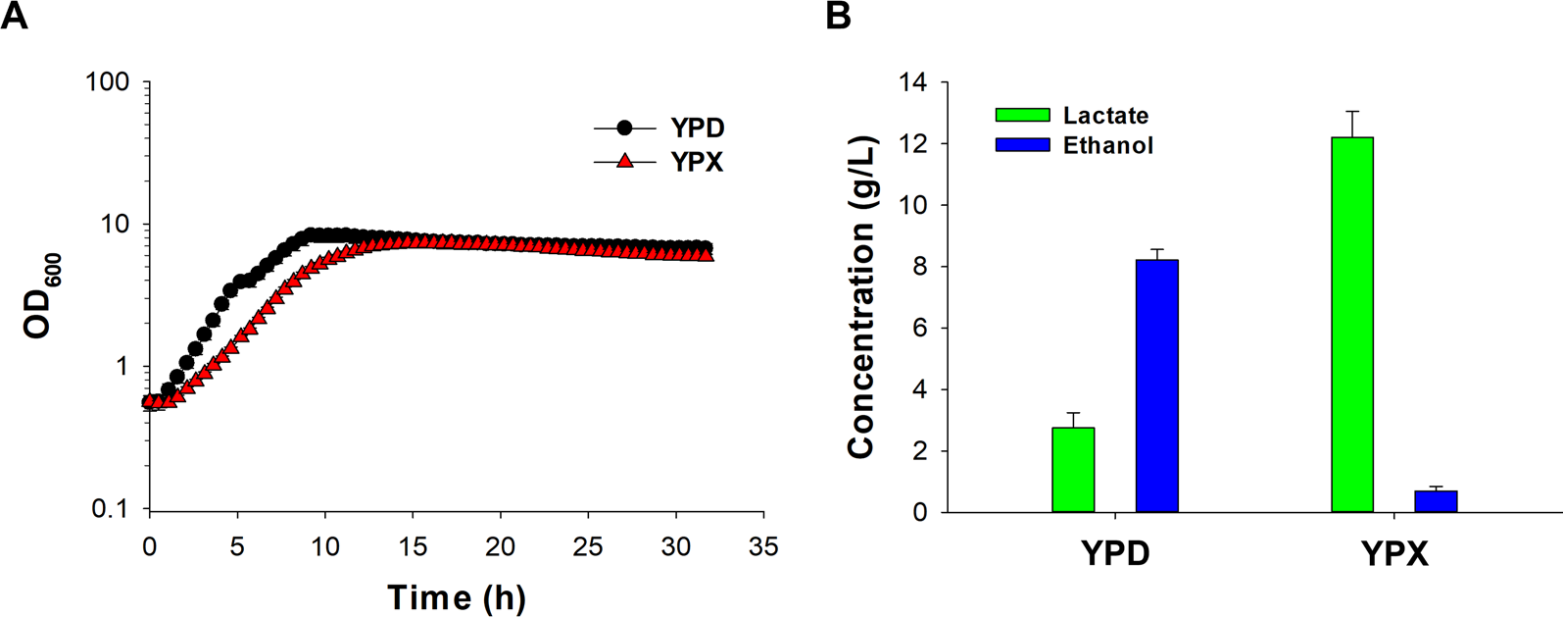
**A** Cultivation of LX3 in YP media containing 20 g/L glucose (YPD) or 20 g/L xylose (YPX) for 32 h. **B** Final titers of lactic acid and ethanol after 32 h fermentation in YPD or YPX media. The experiment was repeated using independent biological triplicates. The averages of the measurements, with error bars representing standard deviations, are shown. Profile of fermentation kinetics is shown in Additional file 1: Fig. S8

After evaluation of the XXX strain as host for lactic acid production, six copies of the *LDH* gene expression modules were integrated into the genome of XXX strain, resulting in the xylose-fermenting and lactic-acid-producing strain LX3. In YPD medium with 20 g/L of glucose, the LX3 strain produced 2.8 ± 0.4 g/L of lactic acid and 8.2 ± 0.2 g/L of ethanol within 32 h (Fig. 2). In YPX medium with 20 g/L xylose, 12.2 ± 0.6 g/L of lactic acid but merely 0.7 ± 0.1 g/L ethanol was produced within 32 h (lactic acid productivity of 0.3 g/L/h). The growth of LX3 rate was significantly higher in glucose (*µ*_max_ of 0.47 h^−1^), compared to xylose containing medium (*µ*_max_ of 0.33 h^−1^).

**Fig. 3:**
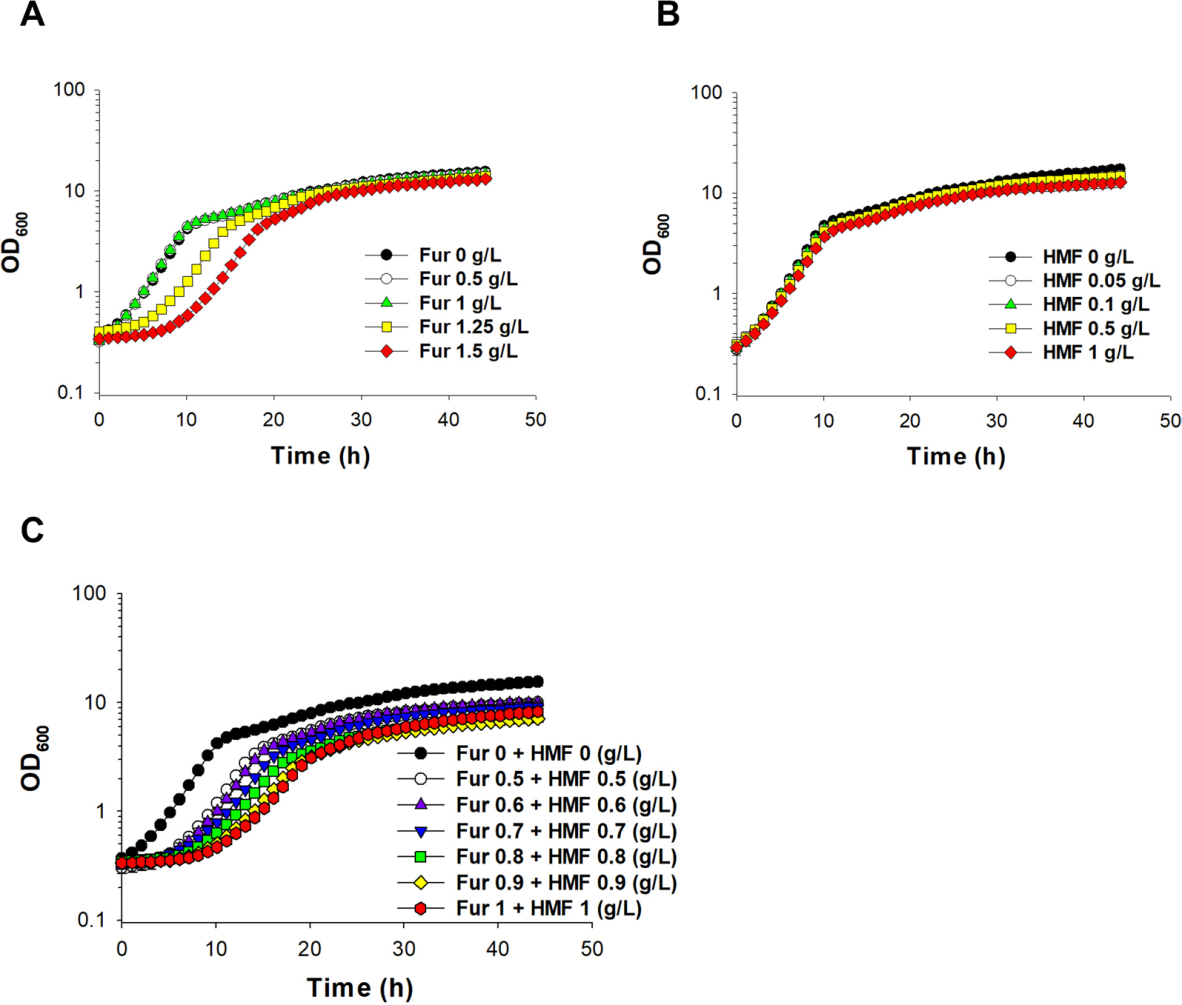
Growth profiles of LX3 in SLH medium supplemented with varying concentrations of **A** furfural, **B** HMF, or **C** a mix of furfural and HMF. The experiment was repeated using independent biological triplicates. The averages of the measurements, with error bars representing standard deviations, are shown

In order to determine the tolerance of LX3 to inhibitors present in lignocellulosic hydrolysate, LX3 was grown in SLH supplemented with furfural (0 to 1.5 g/L), HMF (0 to 1 g/L), or mix of furfural and HMF (0 to 1 g/L of each) for 46 h. LX3 was able to grow in all studied concentrations of furfural, HMF, or mix of thereof (Fig. 3). No difference was observed at the maximum cell density (OD_600_ 13.1 to 15.4) and specific growth rate (*µ*_max_ 0.30 to 0.31 h^−1^) in all concentrations of furfural. However, the lag phases were extended to 4 h in 1.25 g/L and 8 h in 1.5 g/L furfural compared to concentrations under the 1 g/L furfural where LX3 had no noticeable lag phase (Fig. 3A). At the HMF concentrations tested (0 to 1 g/L), no differences in growth profiles were observed (Fig. 3B). The mix of furfural and HMF had an additive effect. Lower concentrations, 0.5–0.7 g/L of furfural and HMF resulted in a 5 h extended lag phase compared to growth without inhibitors and 67% reduction of maximum cell density (OD_600_ of 9.7 for 0.5–0.7 g/L of furfural and HMF, compared to an OD_600_ of 15.6 in control conditions). At higher furfural and HMF concentrations (0.8–1 g/L of each inhibitor), a 49% reduction in maximum cell density (OD_600_ 6.8–8.1), a 7.5 h of extended lag phase, and a 78% reduction of maximum specific growth rate (*µ*_max_ 0.25 to 0.26 h^−1^) were observed.

### Bioconversion of furan-derived inhibitors by *S. cerevisiae* CEN.PK LX3 and ADP1 ASA714

**Fig. 4:**
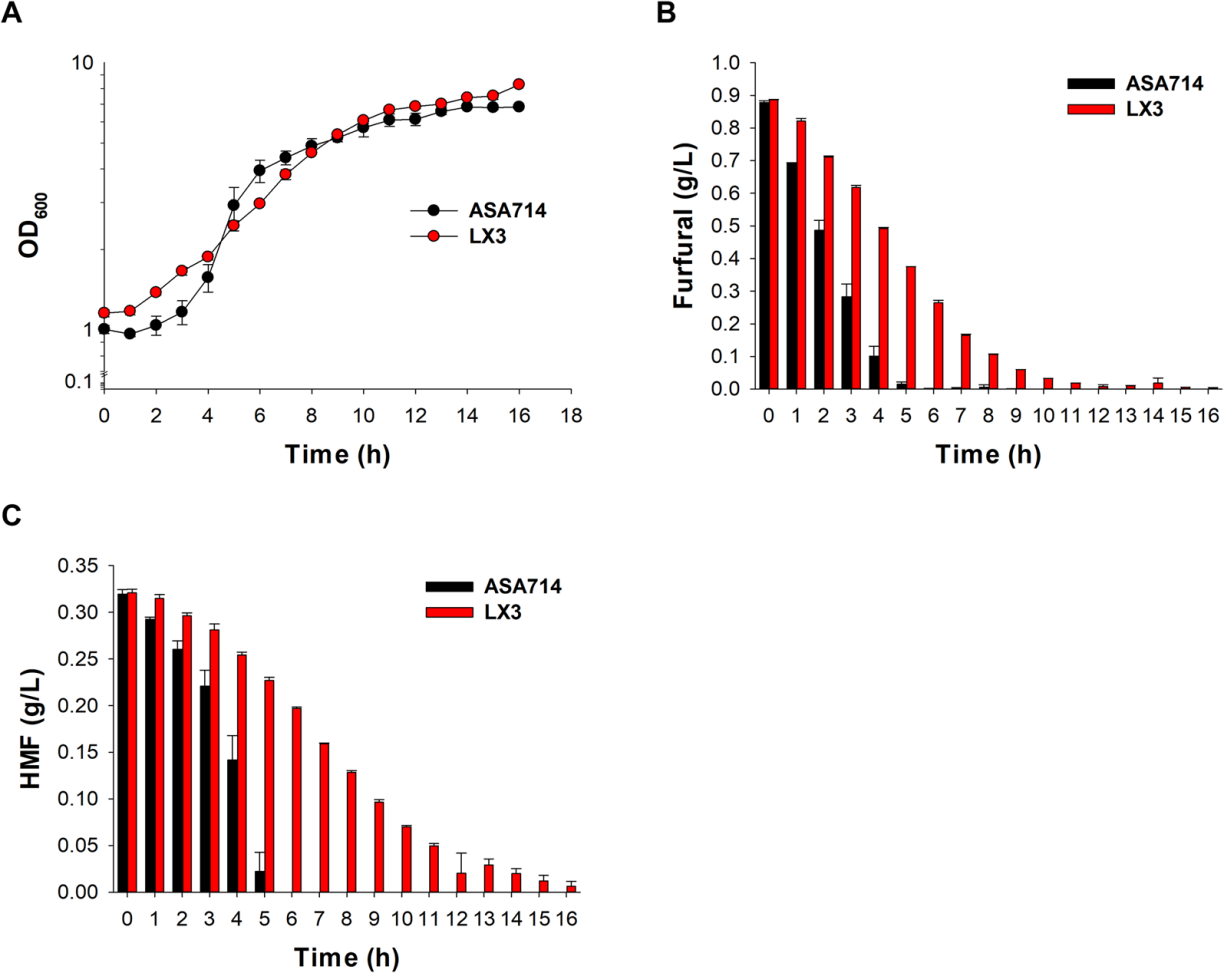
Bioconversion of furfural and HMF by LX3 and ASA714. The cells were grown in SLH medium, and **A** the growth and bioconversion of **B** furfural and **C** HMF were determined. The experiment was repeated using independent biological replicates. The averages of the measurements, with error bars representing standard deviations, are shown

Neither *S. cerevisiae* nor ADP1 can utilize furfural and HMF as carbon sources, but both strains are able to convert them to other furan-derived compounds [11, 22–24]. We, therefore, expected this to occur also in the coculture. To determine the capacity of the engineered strains LX3 and ASA714 to detoxify the furan aldehydes, we cultivated the strains individually in SLH medium and analyzed the consumption of furfural and HMF (Fig. 4). ASA714 showed faster bioconversion of furfural and HMF compared to X3 (Fig. 4, Table 2). Within 6 h, ASA714 completely metabolized the furfural and HMF of the medium, while furfural and HMF were converted by LX3 within 12 and 17 h, respectively. The average furfural and HMF conversion rates of ASA714 were 0.15 g/L/h and 0.05 g/L/h, respectively, which was about 2-fold and 2.8-fold higher than the rates observed for LX3 (Table 2). The total time needed to completely metabolize furfural and HMF by ASA714 was similar, but the conversion rate was found to be growth phase dependent. While the HMF was metabolized during the exponential growth, most furfural was converted already during the lag phase of ASA714. The LX3 strain metabolized all the furfural approximately 5 h faster than HMF, and both furfural and HMF were mainly metabolized during the exponential phase (Fig. 4).

**Table 2:** Rate of bioconversion of furfural and HMF by LX3 and ASA714

Strain	Inhibitor	Conc. (g/L)	Total time required for complete conversion (h)	Average conversion rate (g/L/h)
ASA714	Furfural	0.88 ± 0.01	6	0.15 ± 0.00
	HMF	0.32 ± 0.01	6	0.05 ± 0.00
LX3	Furfural	0.89 ± 0.00	12	0.07 ± 0.00
	HMF	0.32 ± 0.00	17	0.02 ± 0.00

### Fermentation of SLH by a coculture of *S. cerevisiae* and ADP1

The ability of LX3 and ASA714 to detoxify and produce lactic acid and WEs during the fermentation of SLH was studied in four independent bioreactor cultivations. Prior the cultivations, an optimal inoculation ratio of the strains was determined; Three different ratios (ADP1:LX3; 2:1, 1:1, and 1:2) were examined and the 2:1 ratio of ADP1 and LX3 was found to perform the best with 2.6-fold higher lactic acid production compared to the equivalent ratio (1:1) of both microbes (Additional file 1: Fig. S9). The 1:2 inoculation ratio of ADP1 and LX3 resulted in almost identical performance as the monoculture (Additional file 1: Fig. S9). Based on substrate consumption, lactic acid production, and side product (ethanol) formation, an inoculation ratio of 1:2 for LX3 and ASA714 was chosen for the bioreactor experiments.

**Fig. 5:**
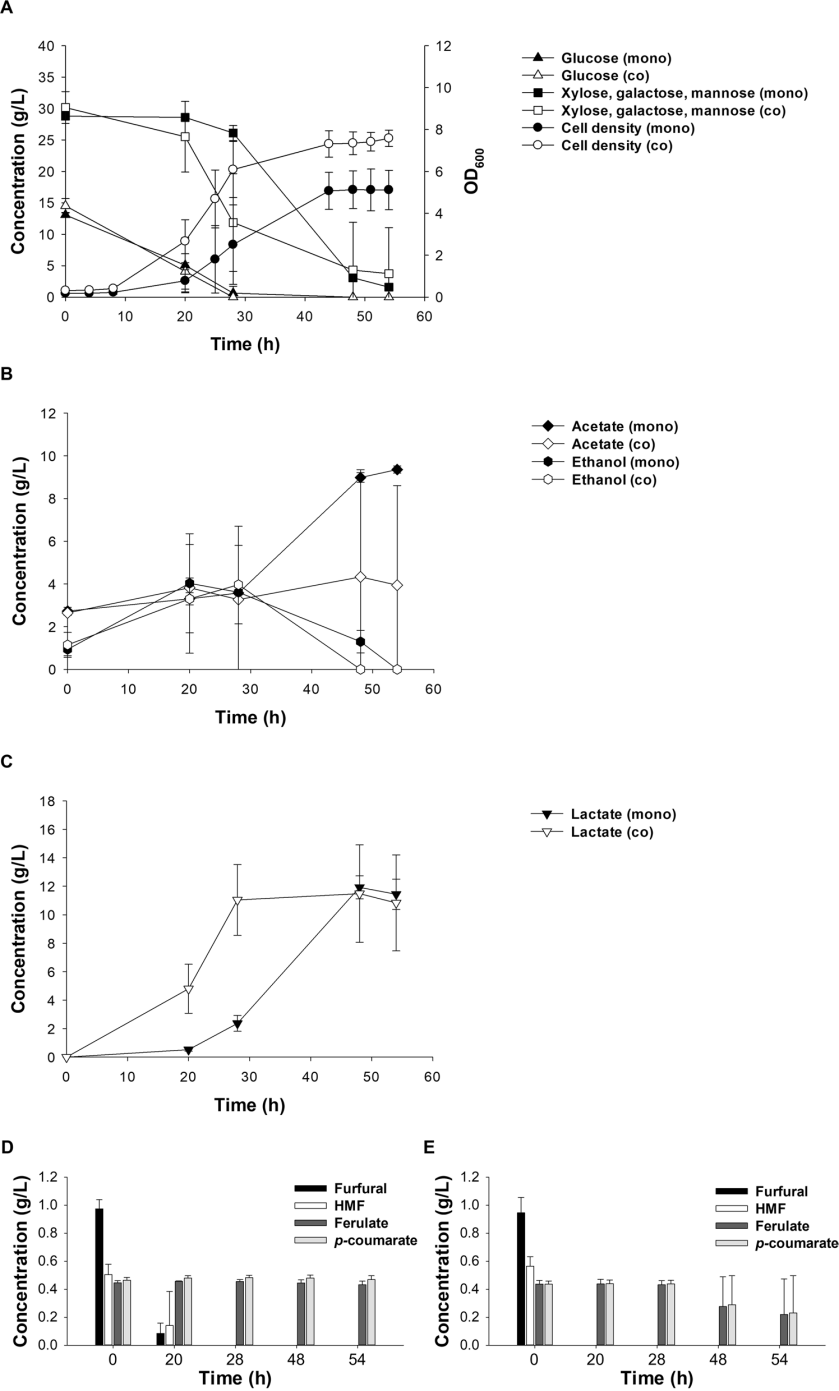
Bioreactor cultivations of LX3 and ASA714. **A**–**C** Metabolites produced and cell growth based on OD_600_ in LX3 monoculture (mono) and LX3-ASA714 coculture (co); concentration of inhibitors in **D** LX3 monoculture and **E** LX3-ASA714 coculture. The experiment was repeated using independent biological quadruplicates (for individual experiments, see Additional file 1: Figs. S10 and S11). The averages of the measurements, with error bars representing standard deviations, are shown

In the coculture, the lag phase was shortened by 16 h and a 1.5 times higher growth rate (*µ*_max_ 0.17 h^−1^) was achieved compared to the monoculture of LX3 (*µ*_max_ 0.11 h^−1^) (Fig. 5A). The glucose consumption was similar in both the monoculture and cocultures, with glucose being depleted within 28 h of fermentation. However, a notable difference was observed in the consumption of the rest of the sugars: in the monoculture, less than 10% of xylose, mannose, and galactose were consumed after 28 h, whereas over 60% consumption of these sugars was observed in the coculture at the same time point (Fig. 5A).

The titer of lactic acid was slightly higher in the coculture (12.0 g/L) compared to the monoculture (11.3 g/L) whereas the yield was 0.3 g_lactic acid_/g_sugars_ for both cultures. Notably, a 1.5 times higher lactic acid productivity was observed in the coculture (0.41 g lactic acid/L/h) compared to that of the monoculture (0.27 g lactic acid/L/h) (Fig. 5C and Table 3). The final concentration of acetic acid in the monoculture was 9.4 g/L compared to that of 3.9 g/L in the coculture.

**Table 3:** Lactic acid productivity, yield, and titer of LX3 monocultures and LX3-ASA714 cocultures on SLH

	Substrate	Lactic acid		
	(g/L)	Productivity (g/L/h)	Yield (g/g)	Titer (g/L)
Monoculture	41.4 ± 0.8	0.27 ± 0.02	0.3 ± 0.03	11.3 ± 1.3
Coculture	44.7 ± 2.2	0.41 ± 0.08	0.3 ± 0.05	12.0 ± 1.8

Ethanol accumulation was similar in both cultures, but in the coculture, the ethanol produced was more rapidly consumed (Fig. 5B). All furfural and HMF were metabolized in both cultures, but at different rates. All the furfural and HMF were metabolized already within 20 h in the coculture, while 0.08 g/L furfural and 0.1 g/L HMF were still detected at the 20 h timepoint in the monoculture (Fig. 5D and E). The phenolic compounds, ferulate and *p*-coumarate, were not consumed in the monocultures whereas an average of 0.2 g/L of each remained after the coculture. Notably, two of the four coculture fermentations showed complete depletion of all acetate and all phenolic compounds, whereas less acetate and no phenolics consumption were observed in the other two cultures (Additional file 1: Figs. S10 and S11).

Wax esters were quantified in cells sampled after 28 h, 48 h, and 54 h of cultivation. Expectedly, no WEs were detected in the LX3 monoculture. Measurable amounts of WEs were only detected in one of the cocultures at the samples taken after 28 h and 48 h (24.4 mg/L and 11.9 mg/L, respectively), whereas in other cocultures, WEs could not be reliably quantified.

## Discussion

Using lignocellulosic biomass as feedstock in bioprocesses is economically and environmentally justified. However, the hydrolysates derived from biomass contain a mixture of different substrates and toxic compounds that can result in growth inhibition of the host microorganism, lowered productivities and yields [52, 53], and sometimes accumulation of unwanted byproducts [54]. For the lignocellulose-based processes to be successful, comprehensive utilization of carbon and the ability to produce a broad range of relevant biochemicals is crucial. Many of the issues could potentially be addressed using a coculture of optimally selected and engineered strains.

In this work, our goal was to establish a coculture for efficient production of lactic acid from a synthetic lignocellulose hydrolysate (SLH) containing xylose, hexoses, aliphatic acids, furan derivatives, and phenolic acids, thus mimicking lignocellulose hydrolysates made from spruce. Lignocellulosic biomass derived from forestry, such as spruce residues, are widely abundant, especially in Northern Europe [55]. Therefore, valorization of lignocellulosic biomass derived from wood has great potential in countries with extensive forest coverage and well-developed forest industries. To further improve the carbon recovery and process feasibility, we aimed to direct the residual carbon and potential byproducts of *S. cerevisiae* toward the production of a high-value product, WEs. To allow efficient carbon recovery in the process, we selected the yeast *S. cerevisiae* and the bacterium *A. baylyi* ADP1 as the host organisms for the coculture. *S. cerevisiae* can efficiently produce lactic acid from major lignocellulose components [56, 57], while ADP1 can metabolize a wide range of organic acids and phenolic compounds that are known to be harmful for *S. cerevisiae* [52], and direct the carbon to secondary products, namely high-value lipids, WEs. While lactic acid can be separated from the cultivation broth using several reported methods for its purification from the fermentation medium [58], WEs accumulate intracellularly and can be extracted from harvested cells, potentially providing an advantage for product recovery.

To engineer ADP1 to be suitable for the coculture, we first characterized wild-type ADP1 for its tolerance to furfural and HMF, as well as its ability to consume lactate. Previously, ADP1 has been shown to grow on acetate in minimal medium with 1 g/L furfural [22]. We confirmed that wild-type ADP1 can tolerate up to 2 g/L furfural and 1.5 g/L HMF when glucose was provided as a carbon source (Additional file 1: Fig. S1). We also demonstrated that ADP1 was able to grow well in SLH and exhibited sufficient tolerance to the mixture of inhibitors present. We further confirmed that wild-type ADP1 naturally utilizes l- and d-lactate, which aligns with previous research [59].

As the starting point for engineering, we selected a previously described strain ASA507 [39]. The strain ASA507 is derived from a genetically stable, transposon-free ADP1-ISx [37] and has exceptionally high tolerance against *p*-coumarate and ferulate [39], the key aromatic monomers of the lignin fraction of lignocellulose. We first removed the complete lactate operon (*lldPRD*, *dld*; ACIAD 0106-0109) encoding a lactate permease, a l-lactate dehydrogenase operon regulator, a l-lactate dehydrogenase, and a d-lactate dehydrogenase. Furthermore, to increase the production of WEs, we overexpressed a fatty acyl-CoA reductase *acr1* (ACIAD 3383), which has been previously shown to improve WE production in ADP1 [45]. We also showed that lactate concentrations relevant to what could be expected from the fermentation of the SLH containing approximately 38 g/L of sugars, e.g., up to 18 g/L lactic acid did not inhibit the growth of ASA714 (Additional file 1: Fig. S5). Lactic acid at a yield of 0.26 g/g was recently reported for *S. cerevisiae* during fermentation of corn stover and wheat straw hydrolysate [60].

ASA714 consumed all acetate, ferulate, and *p*-coumarate in the SLH medium, and as a result of its incomplete sugar metabolism [21], no xylose, galactose, or mannose was consumed (data not shown). Thus, glucose is the only sugar found in the SLH medium that ADP1 potentially competes for with *S. cerevisiae*. Unexpectedly, ASA714 consumed only about 0.7 g/L of glucose in the SLH medium; the same was observed for wild-type ADP1 grown in minimal medium supplemented with 10 g/L glucose and 2.6 g/L acetate (data not shown), demonstrating that the inefficiency of glucose utilization was not a result of strain engineering but a phenotypic feature of ADP1. In fact, ADP1 did not start utilizing glucose even in 3 days after acetate depletion. On the contrary, in a previous study, ADP1 was shown to co-utilize acetate and glucose when grown in minimal medium containing less substrates, e.g., 2 g/L of both acetate and glucose [61]. Moreover, ADP1 has been shown to utilize gluconate concurrently with acetate, even at substrate concentrations as high as 100 mM (5.9 g/L acetate and 19.5 g/L gluconate) [28]. Gluconate is metabolized by the same modified Entner–Doudoroff pathway as glucose [21], so it is not clear why the presence of acetate has such an inhibitory effect on glucose but not on gluconate utilization. Although interesting, the determination of the reason behind the reduced ability to consume glucose of ADP1 in the studied conditions remains to be investigated in the future. In the context of the studied coculture, however, the hampered glucose metabolism in the presence of acetate was found to be beneficial, as ADP1 did not compete for sugars with *S. cerevisiae*. Taken together, the characteristics of ASA714 in terms of tolerance and carbon utilization were found to be well-suited for the coculture.

The lactic-acid-producing strain LX3 was constructed based on the rational design of genetic perturbations to increase the activity of the lactic acid metabolic pathway with introduction of multiple copies of the *LDH* gene and simultaneously deleting *CYB2*, *ERF2*, and *GPD1* in the xylose-fermenting strain, XXX [38]. Deletion of *CYB2*, encoding a lactate cytochrome-c oxidoreductase, has been known to prevent assimilation of lactate at low pH [62], whereas deletion of *ERF2*, which encodes a palmitoyl transferase, enhanced acid tolerance of *S. cerevisiae* [63]. *GPD1*, encoding a glycerol-3-phosphate dehydrogenase, was deleted to increase the lactic acid metabolic pathway flux by reducing the activity of the glycerol pathway [63]. The advantage of lactic acid fermentation from xylose was described in a previous study: when xylose was used as substrate, 28-times less ethanol, the major fermentation product in *S. cerevisiae*, was produced compared to when glucose was used as the substrate [64]. This was also observed in LX3 fermentation. The LX3 strain produced 5.9-times less ethanol from xylose than from glucose. The lactic acid production of LX3 was 4 times higher from xylose than from glucose. Turner et al. [64] proposed that the slower rate of xylose utilization, compared to glucose, prevents the accumulation of an excessive amount of intracellular pyruvate, which typically leads to ethanol production. Instead, this slower utilization rate results in the conversion of pyruvate to lactic acid [64]. These characteristics make the LX3 strain not only an ideal host for carbon utilization in lignocellulosic hydrolysates, but also an efficient producer of lactic acid.

The aerobic biotransformation of furfural and HMF to their corresponding alcohols (and to some extent, furfural to furoic acid) by *S. cerevisiae* has been previously reported [65, 66]. Despite the ability of *S. cerevisiae* to detoxify furan derivatives, the presence of these compounds has been shown to interrupt the performance of *S. cerevisiae* already in the early stages of fermentations [67]. In addition, furfural has been shown to negatively affect glycolysis [68] and both HMF and furfural can cause a prolonged lag phase of *S. cerevisiae* [66, 69, 70]. Besides inhibiting cell growth, previous research has demonstrated that furfural and HMF can directly negatively impact bioproduction, such as ethanol production [71]. While acetic acid is toxic to *S. cerevisiae* on its own [72], it has also been reported to increase the toxicity of furfural to *S. cerevisiae* [73]*.* Species of *Acinetobacter* genus have been also described to metabolize furfural and HMF [22–24]. To investigate whether coculturing the strains would accelerate the detoxification and reduce the inhibitory effect of furan-derived compounds on *S. cerevisiae*, we first studied the bioconversion capacity of both cocultivation partners individually. LX3 converted furfural and HMF in 12 h and 17 h (conversion rate of furfural 0.07 g/L/h and HMF 0.02 g/L/h). Previously, *S. cerevisiae* was reported to convert furfural and HMF with a higher conversion rate (furfural 0.14 g/L/h and HMF 0.27 g/L/h; estimated from the figures) [74] compared to what we measure for LX3; the conversion rate is likely to be strain and condition dependent. ASA714 converted all furfural and HMF within 6 h. Previously, ADP1 was shown to convert 1 g/L furfural (in a minimal medium with acetate as the sole carbon source) [22] slightly faster than in our experiments. This is likely due to the complex nature and combined inhibitors in the SLH. Taken together, the detoxification studies demonstrated that ADP1 has potential to serve as an efficient detoxifier of lignocellulosic hydrolysates and can, thus, potentially support *S. cerevisiae* growth and production process.

For a coculture to strive, not only the choice of microorganisms but also the inoculation ratio needs to be considered [75]. Therefore, prior conducting the coculture of ASA714 and LX3 with SLH medium, the effect of initial inoculation ratio was investigated. The improved coculture performance seen with increasing the proportion of ADP1 (to 2:1) can be explained by ADP1’s more prominent role in detoxification, given its high furan derivative bioconversion rate. A similar trend was previously demonstrated in a coculture with a filamentous fungus and a yeast: a higher inoculation size of *Fusarium striatum* that converted furfural and HMF in a lignocellulosic hydrolysate resulted in more efficient production of ethanol by *S. cerevisiae* in their coculture [76].

Based on the initial strain characterizations, it is likely that LX3 consumed most of the sugars also in the coculture. Hexoses probably contributed mainly to the biomass accumulation of LX3 while the xylose was primarily converted to lactic acid. Notably, sugar consumption was faster in the coculture compared to the monoculture of LX3 (Fig. 4). Ethanol production was observed both in monoculture and cocultures (Fig. 5B). We observed that ethanol was depleted faster in the coculture than in the monoculture, probably due to the metabolic activity of ASA714; ethanol is one of the most preferred carbon sources for ADP1 [61]. ADP1 converts ethanol to acetaldehyde, followed by conversion of acetaldehyde to acetate. Consequently, acetate was first generated from ethanol before it was reassimilated by ADP1 (Additional file 1: Fig. S12). Even though the acetate concentration measured by HPLC did not decrease in the coculture, the acetate produced from ethanol (that was depleted) was probably consumed simultaneously as it was produced by ASA714. While some acetate remained in the coculture, the acetate concentration was lower compared to that of the monoculture. Thus, it can be concluded that both substrates and byproducts (acetate and ethanol) of *S. cerevisiae* could be more effectively utilized in the coculture.

The monoculture and coculture experiments were repeated four times (for the data of individual experiments, see Additional file 1: Figs. S10 and S11). There were batch to batch variations in the consumption of acetate and phenolic compounds and the production of WEs among the repetitions. In the 1st and 4th experiment, all acetate was depleted by the 28-h timepoint, followed by the consumption of the phenolic compounds. While it has been previously reported that *S. cerevisiae* has the ability to convert phenolic compounds into less toxic derivatives, consumption of ferulate and *p*-coumarate during the mono-cultivations of LX3 did not occur. Therefore, we assume that ASA714 was solely responsible for the consumption of the phenolic compounds via the 3-ketoadipate pathway, as was demonstrated earlier [19, 20]. In the 2nd and 3rd repletion of the cocultures no phenolic acids were consumed. We reason that this may be due to the higher concentration of acetate in these cultures, which serves as a carbon catabolite repressor for the aromatics degradation pathway [20]. Furthermore, the differences in carbon consumption (Additional file 1: Fig. S11) and WE production profiles among the replicate cultures indicate that the population ratios vary in time across the batches. This could be expected as we did not have any means for population control during the cocultures. A small change in the performance of either coculture partner at the beginning of the culture due to, for instance, decreased viability or fitness could affect the population dynamics during the entire cultivation. The interaction in our bacterium-yeast consortium of ADP1 and *S. cerevisiae* can be classified as facultative mutualism, in which both strains are able to independently grow in the SLH medium.

ASA714 was able to successfully direct some of the carbon, likely from acetate and phenolic acids, toward WE production, despite the process conditions in the coculture being far from optimal for lipid accumulation. In contrast, no WEs were detected from LX3 monocultures, which is consistent with the previous studies showing that *S. cerevisiae* does not naturally produce WEs [77]. ADP1 natively produces WEs mainly in conditions with high carbon–nitrogen ratio [78], whereas the process conditions used in this study exhibited a relatively low carbon–nitrogen ratio. The high nitrogen content directs substrates toward biomass production and more importantly, can drive the cells to rapidly consume the accumulated WEs as the carbon source [50], which likely also explains why some WEs were lost over time (24.4 mg/L at 28 h and 11.9 mg/L at 48 h). In addition, based on the carbon consumption data and visual observation of produced mScarlet fluorescence protein (this dyed the cells of ASA714 to pink), the number of ASA714 cells was likely not very high in the cocultures, emphasizing the difficulty to achieve high WEs titers. We have previously described an autonomous switch for WE accumulation in low carbon conditions [26], as well as improved WE production in nitrogen-rich conditions [44] and by partitioning metabolism for strict control of biomass and WE production [28]. Adapting these approaches in addition to overexpression of the WE synthesis pathway could have further enhanced the WE production metrics, but on the other hand, may have potentially compromised the robustness of the production strain.

The greatest benefit of the coculture was observed in the production of lactic acid. The productivity of lactic acid was improved 1.5-fold (to 0.41 ± 0.08 g/L/h) compared to that of the monoculture. This report presents the first instance of using coculture to improve production of lactic acid by *S. cerevisiae* in a lignocellulosic feedstock. Based on the cell growth and sugar consumption during the first 28 h of the coculture, LX3 grew faster and to a higher biomass concentration in the coculture, which we assume was due to the rapid detoxification of the inhibitors in the early stages of fermentation by ASA714. Despite sharing some of the resources in the culture, such as oxygen and nitrogen source, it is worth noting that the titer and yield of lactic acid were not negatively affected by potential coculture interactions or competition for resources. Taken together, the improved cell growth, carbon recovery, and productivity demonstrate the compatibility of *S. cerevisiae* and *A. baylyi* ADP1 and the potential of cocultures for upgrading complex and heterogeneous substrates.

## Conclusion

In this study, our aim was to increase the efficiency and feasibility of lactic acid production by *S. cerevisiae* using a lignocellulosic feedstock. Toward that end, we established a synthetic microbial consortium employing a strictly aerobic soil bacterium ADP1 with *S. cerevisiae*. ADP1 exhibited high bioconversion rate on furan derivatives in the lignocellulosic hydrolysate, which supported *S. cerevisiae*’s growth and lactic acid production. With the synthetic microbial consortium, the productivity of lactic acid was increased 1.5-fold. No competition for sugars between *S. cerevisiae* and ADP1 occurred in the coculture, allowing for sustained lactic acid titer and yield. In addition, ADP1 consumed residual carbon in the coculture and directed it to high-value lipid (WE) production. The work demonstrates the potential of cocultures for the production of biochemicals from complex and challenging feedstock, such as lignocellulose hydrolysates.

### Supplementary Information

**Additional file 1: Table S1.** Primers used in this study. **Table S2.** Plasmids used in this study. **Figure S1.** Growth curve of the wild-type ADP1 in mineral salts medium (MSM) supplemented with 10 g/L glucose and (A) furfural or (B) HMF at concentration of 0, 0.25, 0.5, 1, 1.5, and 2 g/L furfural or HMF. An overnight pre-culture was carried out in 5 ml MSM supplemented with 4 g/L glucose, 2 g/L casein amino acid, 0.1 g/L furfural, 0.1 g/L HMF, at 30 °C, 300 rpm. The main culture was carried out using 96-well plate, 200 µL cultivation, Spark multimode microplate reader (Tecan, Switzerland), at 30 °C. The experiment was repeated using independent biological triplicates, and the averages of the measurements, with error bars representing standard deviations are shown. **Figure S2.** Growth curve of wild-type ADP1 in SLH medium. An overnight pre-culture was carried out in 5 mL modified SLH medium, at 30 °C, 300 rpm. The main culture was carried out using 96-well plate, 200 µL cultivation, Spark multimode microplate reader (Tecan, Switzerland), at 30 °C. The experiment was repeated using independent biological triplicates, and the averages of the measurements, with error bars representing standard deviations are shown. **Figure S3.** Growth curve of wild-type ADP1 in MSM supplemented with 50 mM lactate as the sole carbon source. An overnight pre-culture was carried in 5 mL MSM supplemented with 10 g/L glucose, 2 g/L casein amino acid, at 30 °C, 300 rpm. The main culture was carried out using 96-well plate, 200 µL cultivation, Spark multimode microplate reader (Tecan, Switzerland), at 30 °C. The experiment was repeated using independent biological triplicates, and the averages of the measurements, with error bars representing standard deviations are shown. **Figure S4.** Visualization of WE accumulation of strains ADP1-ISx and ASA714 using thin layer chromatography (TLC). The cultivations were carried out at 30 °C, 300 rpm, using 100 mL Erlenmeyer flask with 10 mL MSM supplemented with 200 mM glucose, 300 rpm*.* The lipid extraction and the semi-quantitative analysis of WEs by TLC were done as described by Santala et al. Biomass samples were normalized to an OD_600_ of 20 of which 30 µL samples were applied on the TLC plate. TLC was carried out using Glass HPTLC Silica Gel 60F_254_ plates (Merck, USA) with a mobile phase of hexane: diethyl ether: acetic acid 90: 15: 1 (v/v/v). Wax esters on the TLC plates were visualized using iodine staining and using jojoba oil as the standard. **Figure S5.** Growth of ASA714 in SLH medium at varying lactate concentrations. The experiment was repeated using independent biological triplicates. An overnight pre-culture was carried in 5 mL SLH medium at 30 °C, 300 rpm. The main culture was carried out using 96-well plate, 200 µL cultures with SLH medium supplemented with 0–18 g/L lactate, Spark multimode microplate reader (Tecan, Switzerland), at 30 °C. The averages of the measurements, with error bars representing standard deviations are shown. Error bars are present but may be obscured by data symbols. **Figure S6.** (A) Growth profiles of the XXX strain in YP media containing 20 g/L glucose, 10 g/L glucose and 10 g/L xylose or 20 g/L xylose for 24 h. (B) Maximum ethanol concentration in each media after 9 h fermentation. The experiment was repeated using independent biological triplicates, and the averages of the measurements, with error bars representing standard deviations are shown. **Figure S7.** Growth profiles of the XXX strain in YPD medium supplemented with lactic acid at concentrations of 0, 5, 10, 30, 50, 100, 250, 500, 750, and 1000 mM lactic acid for 72 h. The experiment was repeated using independent biological triplicates, and the averages of the measurements, with error bars representing standard deviations are shown. **Figure S8.** Fermentation kinetics of LX3 in (A) YPD or (B) YPX media for 32 h. The experiment was repeated using independent biological triplicates. The averages of the measurements, with error bars representing standard deviations are shown. **Figure S9.** Fermentation kinetics including profiles of (A) cell growth, (B) sugar consumption, (C) lactate, (D) acetate, and (E) ethanol production of LX3 monoculture and coculture of LX3 and ADP1 at inoculation ratio of 1:2, 1:1, and 2:1. Fermentations were carried out in a 3.5 L bioreactor with 1 L SLH medium in 30 °C and pH was maintained at 6.0 by 2 M HCl and 8% (*v/v*) NH_4_OH solutions. **Figure S10.** Fermentation kinetics of (A) 1st, (B) 2nd, (C) 3rd, and (D) 4th LX3 monoculture in a bioreactor. Fermentations were carried out in a 3.5 L bioreactor with 1 L SLH medium at 30 °C and pH was maintained at 7.0 by 2 M HCl and 8% (*v/v*) NH_4_OH solutions. **Figure S11.** Fermentation kinetics of (A) 1st, (B) 2nd, (C) 3rd, and (D) 4th LX3 and ASA714 coculture in a bioreactor. Fermentations were carried out in a 3.5 L bioreactor with 1 L SLH medium at 30 °C and pH was maintained at 7.0 by 2 M HCl and 8% (v/v) NH_4_OH solutions. **Figure S12.** Growth and consumption of carbon sources by ASA714 in MSM supplemented with 8 g/L ethanol, 2.6 g/L acetate, and 2 g/L glucose. The cultivations were carried out using 100 mL Erlenmeyer flask with 10 mL cultivations, at 30 °C and 200 rpm*.* The experiment was repeated using independent biological replicates, and the averages of the measurements, with error bars representing standard deviations are shown.

## Data Availability

The data that support the findings of this study are included within the article or the additional files. The corresponding author is willing to provide the raw data related to this manuscript upon reasonable request.

## Abbreviations

ADP1: *Acinetobacter baylyi* ADP1
ATP: Adenosine triphosphate
CRISPR-ERA: Clustered regularly interspaced short palindromic repeats-mediated editing, repression, and activation
HMF: 5-Hydroxymethylfurfural
HPLC: High-performance liquid chromatography
LB: Lysogeny broth
NAD(P)H: Nicotinamide adenine dinucleotide (phosphate) oxidase
NMR: ^1^H nuclear magnetic resonance
OD_600_: Optical density at 600 nm
PCR: Polymerase chain reaction
PLA: Polylactic acid
PDA: Photodiode array
SLH: Synthetic lignocellulosic hydrolysate
WE: Wax ester
YP: Yeast peptone
