# Supplementary material for: Enhanced upgrading of lignocellulosic substrates by coculture of Saccharomyces cerevisiae and Acinetobacter baylyi ADP1
Source: Biotechnol Biofuels Bioprod. 2024 May 6;17:61. doi: 10.1186/s13068-024-02510-8 (PMC11075230; doi:10.1186/s13068-024-02510-8)
Supplement: Supplementary file 1 — Additional file 1: Table S1. Primers used in this study. Table S2. Plasmids used in this study. Figure S1. Growth curve of the wild-type ADP1 in mineral salts medium (MSM) supplemented with 10 g/L glucose and (A) furfural or (B) HMF at concentration of 0, 0.25, 0.5, 1, 1.5, and 2 g/L furfural or HMF. An overnight pre-culture was carried out in 5 ml MSM supplemented with 4 g/L glucose, 2 g/L casein amino acid, 0.1 g/L furfural, 0.1 g/L HMF, at 30 °C, 300 rpm. The main culture was carried out using 96-well plate, 200 µL cultivation, Spark multimode microplate reader (Tecan, Switzerland), at 30 °C. The experiment was repeated using independent biological triplicates, and the averages of the measurements, with error bars representing standard deviations are shown. Figure S2. Growth curve of wild-type ADP1 in SLH medium. An overnight pre-culture was carried out in 5 mL modified SLH medium, at 30 °C, 300 rpm. The main culture was carried out using 96-well plate, 200 µL cultivation, Spark multimode microplate reader (Tecan, Switzerland), at 30 °C. The experiment was repeated using independent biological triplicates, and the averages of the measurements, with error bars representing standard deviations are shown. Figure S3. Growth curve of wild-type ADP1 in MSM supplemented with 50 mM lactate as the sole carbon source. An overnight pre-culture was carried in 5 mL MSM supplemented with 10 g/L glucose, 2 g/L casein amino acid, at 30 °C, 300 rpm. The main culture was carried out using 96-well plate, 200 µL cultivation, Spark multimode microplate reader (Tecan, Switzerland), at 30 °C. The experiment was repeated using independent biological triplicates, and the averages of the measurements, with error bars representing standard deviations are shown. Figure S4. Visualization of WE accumulation of strains ADP1-ISx and ASA714 using thin layer chromatography (TLC). The cultivations were carried out at 30 °C, 300 rpm, using 100 mL Erlenmeyer flask with 10 mL MSM supplemented with 20 [file 13068_2024_2510_MOESM1_ESM.docx]

**Additional file 1**

**Enhanced upgrading of lignocellulosic substrates by coculture of *Saccharomyces cerevisiae* and *Acinetobacter baylyi* ADP1**

Changshuo Liu^1a^, Bohyun Choi^2a^, Elena Efimova^1^, Yvonne Nygård^2,3^, Suvi Santala^1*^

^1^ Faculty of Engineering and Natural Sciences, Hervanta Campus, Tampere University, Tampere, Finland

^2^ Department of Life Sciences, Industrial Biotechnology, Chalmers University of Technology, Gothenburg, Sweden

^3^ VTT Technical Research Centre of Finland, Espoo, Finland

^a^ The authors contributed equally to this work.

Changshuo Liu: [changshuo.liu@tuni.fi](mailto:changshuo.liu@tuni.fi) (ORCID: 0009-0001-1544-3694)

Bohyun Choi: [bohyun@chalmers.se](mailto:bohyun@chalmers.se) (ORCID: 0000-0001-5547-3911)

Elena Efimova: [elena.efimova@tuni.fi](mailto:elena.efimova@tuni.fi)

Yvonne Nygård: [yvonne.nygard@vtt.fi](mailto:yvonne.nygard@vtt.fi) (ORCID: 0000-0001-6117-0343)

Suvi Santala: [suvi.santala@tuni.fi](mailto:suvi.santala@tuni.fi) (ORCID: 0000-0002-0047-5319)

^*^Corresponding author: Suvi Santala (suvi.santala@tuni.fi)

**Table S1.** Primers used in this study.

| **Purpose** | **Template** | **ID** | **Description** | **Sequences (5’ to 3’)** |
| --- | --- | --- | --- | --- |
| Amplification of the genes *tdk/kan^R^* for *lldPRD,dld* knock-out cassette | ADP1 *Δacr1*::*tdk/kanR* [1] | CL_22_05 | Fw | CCCAGCCTCCAATTCAAATCAT |
|  |  | CL_22_06 | Rv | CCAGCTCCGCATGCTTAGAA |
| Amplification of the upstream flanking sequence for *lldPRD,dld* markerless rescue cassette (contains MfeI, NotI, AvrII) using SOE PCR | Wild-type ADP1 | CL_22_11 | Fw | TTCACCATTTGCCATCTGATAACTGG |
|  |  | CL_22_12 | Rv | CCTAGGGCGGCCGCCAATTGTTTCATCTCCTTGAAATGCCGATG |
| Amplification of the downstream flanking sequence for *lldPRD,dld* markerless rescue cassette (contains MfeI, NotI, AvrII) using SOE PCR | Wild-type ADP1 | CL_22_13 | Fw | CAATTGGCGGCCGCCCTAGGCAGACCAAAACATAATACAAAAATACATAGC |
|  |  | CL_22_14 | Rv | AGCATTCTGATCGTTTACACAGC |
| Amplification of the upstream flanking sequence for *lldPRD,dld* knock-out (contains an overhang sequence for *tdk/kan^R^*) using SOE PCR | Wild-type ADP1 | CL_22_15 | Rv | ATGATTTGAATTGGAGGCTGGGTTTCATCTCCTTGAAATGCCGATG |
| Amplification of the downstream flanking sequence for *lldPRD,dld* knock-out, (contains an overhang sequence for *tdk/kan^R^*) using SOE PCR | Wild-type ADP1 | CL_22_16 | Fw | TTCTAAGCATGCGGAGCTGGCAGACCAAAACATAATACAAAAATACATAGC |
| Verification of *lldPRD,dld* knock-out | ASA711, 714 | CL_22_17 | Fw | TGTGAGAACAGTTTCTGCTGA |
|  |  | CL_22_18 | Rv | TTCATCTTCTCCAACATACTGACC |
| Amplification of the *acr1* knock-out and overexpression cassettes | ADP1 Acr1 [2] and ADP1 ASA523 [3] | JL_20_10 | Fw | TTAAAGCAGCAGGCGTTTCAGA |
|  |  | JL_20_15 | Rv | TGCTGTTTGATGTGGGCATTG |
| Verification of *acr1* knock-out | ASA711, 714 | tl50 | Fw | ACGACCACGCATCGTCAGC |
|  |  | tl38 | Rv | TGCTTGGGCCACTCATGC |
| Verification of *poxB* knock-out | ASA714 | 3381v1 | Fw | TTGGCTAACTTGTCAAAGTC |
|  |  | 3381v2 | Rv | TAGAGTGTAGAAACAGATGC |
| Amplification upstream *pTDH3-ldh-tCYC1* cassette to replace CYB2 | YIP01 | BC01 | Fw | CCGCTACTAGTTCCGTGGCGTATCTAAACTGGCATAATGGCCAAATAGACCGGATAACAATTTCACACAG |
|  |  | BC02 | Rv | GGCTTGTTATGCTTGGCAACTTCAGCGGGCGAAATCTTTTGTTTATTCATCAAGGCGATTAAGTTGGGT2 |
| Amplification upstream *pTDH3-ldh-tCYC1* cassette to replace ERF2 | YIP01 | BC09 | Fw | CATACAGGAGAAGGTTCATTGACGAAGTTGTTCTTCCGATGGCTTGTTACCGGATAACAATTTCACACAG |
|  |  | BC10 | Rv | AGGAAGAATATATAATTCGAGACATTCGGTAACGATATATAACCTTTTCCCAAGGCGATTAAGTTGGGT |
| Amplification upstream *pTEF2-ldh-tCYC1* cassette to replace GPD1 | YIP02 | BC27 | Fw | AAAAATTGACTGAAATCATAAATACTAGACATCAAAACGTGAAATACTTGCACACAGGAAACAGCTATG |
|  |  | BC28 | Rv | GTTGAAAACGATGATGTCGACATCCTTGACTGAATCAATCAAGTCTGGATCAAGGCGATTAAGTTGGGT |
| sgRNA for CYB2 | na | BC03 | Fw | GACTAATAGACAACGAGCCGAAAC |
|  |  | BC04 | Rv | AAACGTTTCGGCTCGTTGTCTATT |
| sgRNA for ERF2 | na | BC11 | Fw | GACTAAATTGTGGCATACCCAAAA |
|  |  | BC12 | Rv | AAACTTTTGGGTATGCCACAATTT |
| sgRNA for GPD1 | na | BC29 | Fw | GACTTGGATTAGCAACCAAATTGT |
|  |  | BC30 | Rv | AAACACAATTTGGTTGCTAATCCA |
| Verification of CYB2 | Colony PCR to verify replacement of *CYB2* with *ldh* expression module | BC07 | Fw | CGTCTAAGACTAGATTGAACA |
|  |  | BC08 | Rv | TTCCCGGCATTAAACTTGATA |
| Verification of ERF2 | Colony PCR to verify replacement of *ERF2* with *ldh* expression module | BC15 | Fw | ACAAGATCGGAAAGCACCT |
|  |  | BC16 | Rv | GCTGTGTGCTGGCGATA |
| Verification of GPD1 | Colony PCR to verify replacement of *GPD1* with *ldh* expression module | BC33 | Fw | TAAACTTAACTTCCGGCCA |
|  |  | BC34 | Rv | TTCAATCATGTCCGGCAG |

**Table S2.** Plasmids used in this study.

| **Plasmid** | **Description** | **Antibiotic resistance** | **Source** |
| --- | --- | --- | --- |
| YIP01 | *pTDH3-ldh-tCYC1* | AmpR | This study |
| YIP02 | *pTEF2-ldh-tCYC1* | AmpR | This study |
| YN2-1 | *pPGK1-Cas9-tPGK1, pSNR52-sfGFP-tSNR52* | AmpR, KanR | [4] |
| BC01 | *pPGK1-Cas9-tPGK1, pSNR52-sgRNA-CYB2-tSNR52* | AmpR, KanR | This study |
| BC02 | *pPGK1-Cas9-tPGK1, pSNR52- sgRNA-ERF2-tSNR52* | AmpR, KanR | This study |
| BC03 | *pPGK1-Cas9-tPGK1, pSNR52- sgRNA-GPD1-tSNR52* | AmpR, KanR | This study |
| mScarlet Gene Cassette | *puc57-poxB flanking-mScarlet-cmR* | AmpR, CmR | [5] |

**
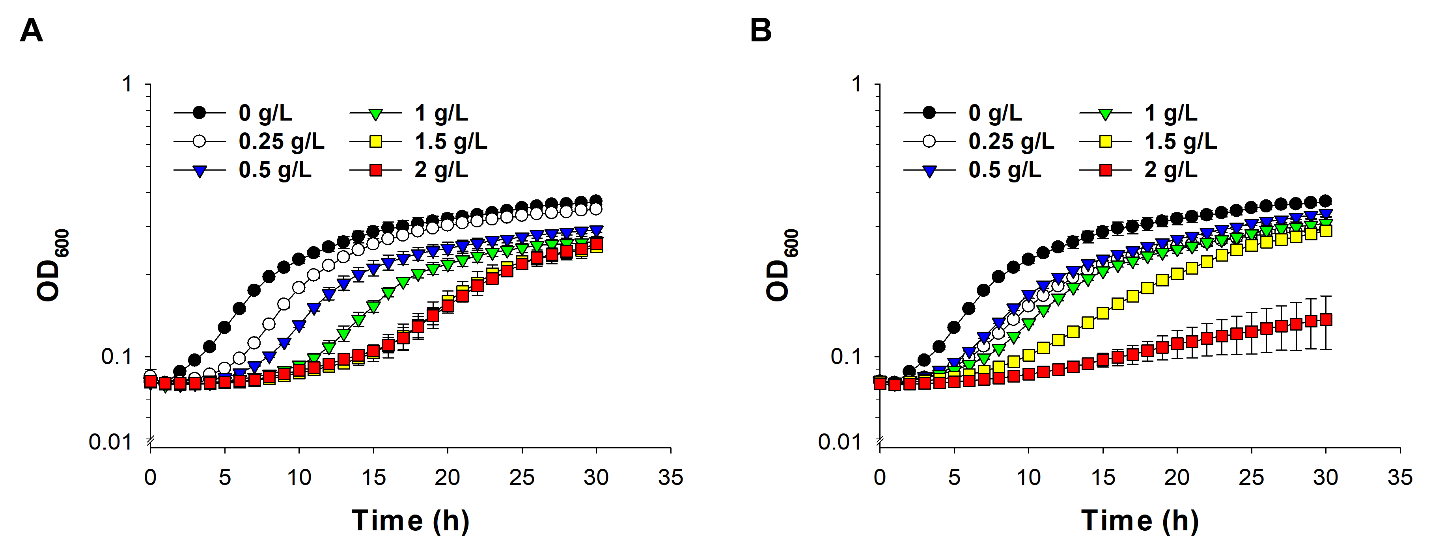
**

**Figure S1.** Growth curve of the wild-type ADP1 in mineral salts medium (MSM) [6] supplemented with 10 g/L glucose and (A) furfural or (B) HMF at concentration of 0, 0.25, 0.5, 1, 1.5, and 2 g/L furfural or HMF. An overnight preculture was carried out in 5 ml MSM supplemented with 4 g/L glucose, 2 g/L casein amino acid, 0.1 g/L furfural, 0.1 g/L HMF, at 30 °C, 300 rpm. The main culture was carried out using 96-well plate, 200 µL cultivation, Spark multimode microplate reader (Tecan, Switzerland), at 30 °C. The experiment was repeated using independent biological triplicates, and the averages of the measurements, with error bars representing standard deviations are shown.


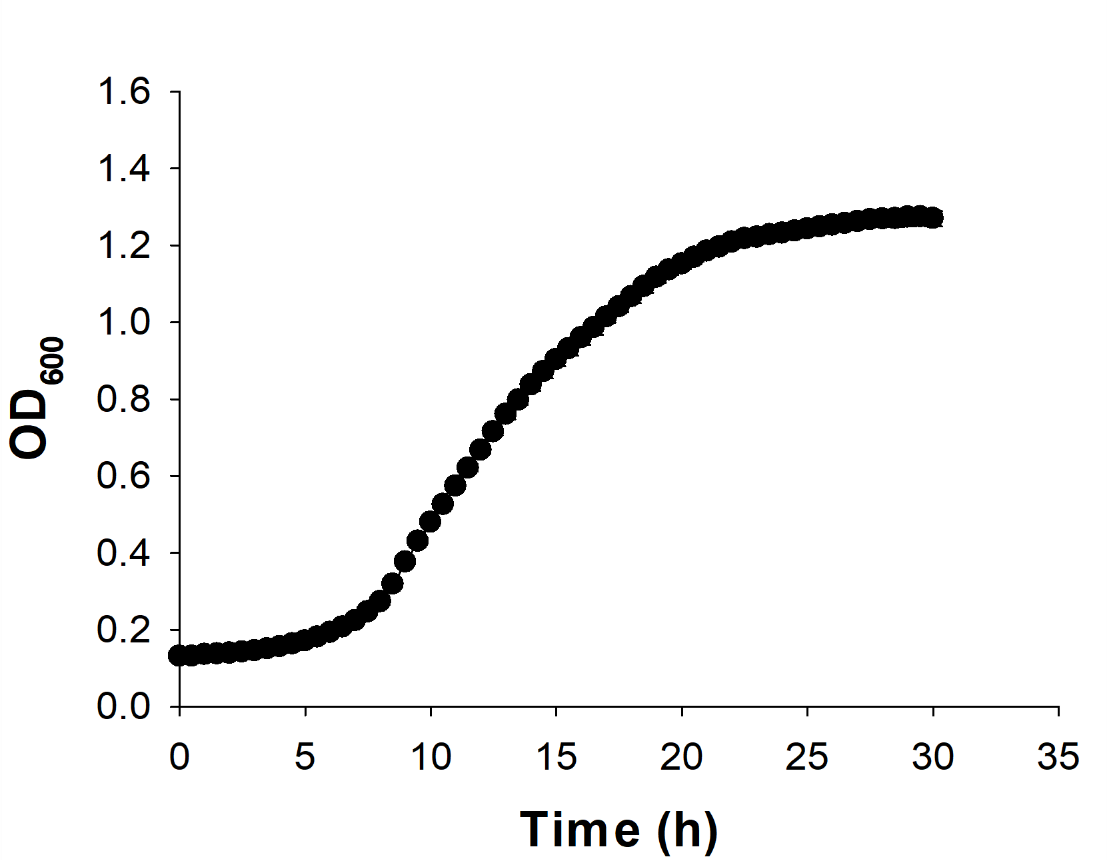


**Figure S2.** Growth curve of wild-type ADP1 in SLH medium. An overnight preculture was carried out in 5 mL modified SLH medium, at 30 °C, 300 rpm. The main culture was carried out using 96-well plate, 200 µL cultivation, Spark multimode microplate reader (Tecan, Switzerland), at 30 °C. The experiment was repeated using independent biological triplicates, and the averages of the measurements, with error bars representing standard deviations are shown.


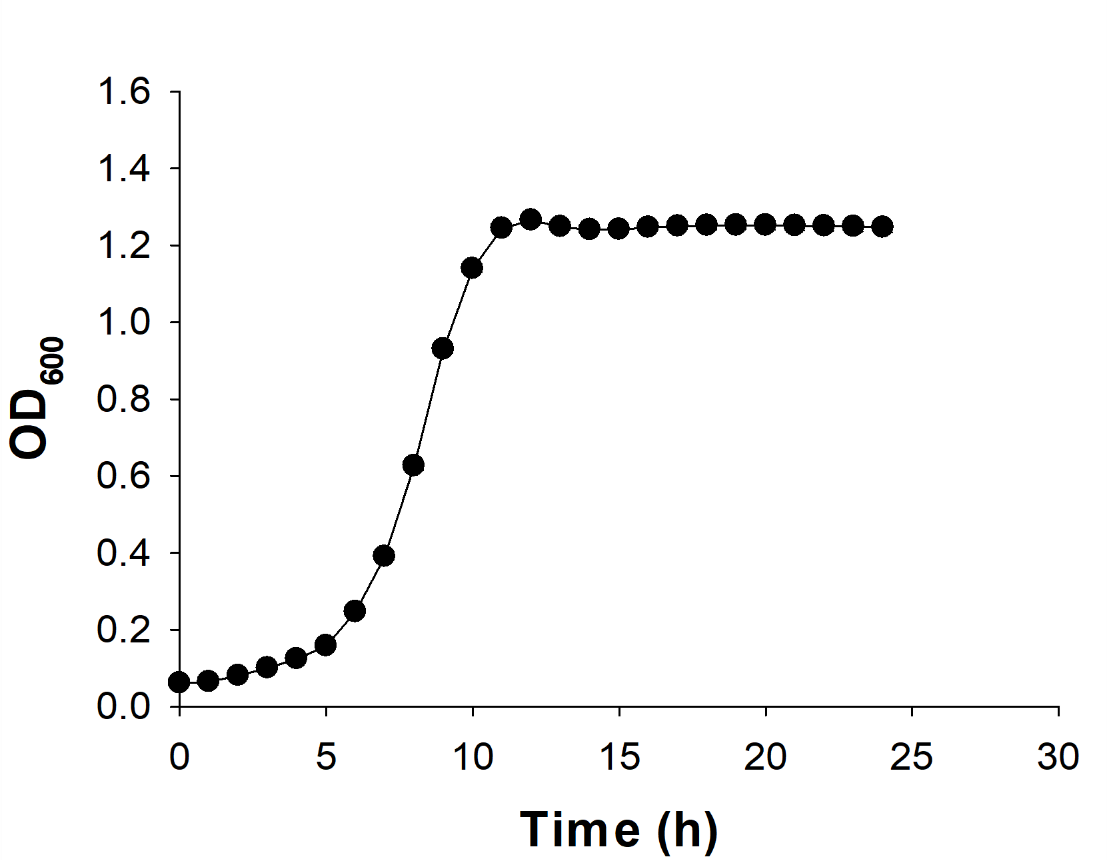


**Figure S3.** Growth curve of wild-type ADP1 in MSM supplemented with 50 mM lactate as the sole carbon source. An overnight preculture was carried in 5 mL MSM supplemented with 10 g/L glucose, 2 g/L casein amino acid, at 30 °C, 300 rpm. The main culture was carried out using 96-well plate, 200 µL cultivation, Spark multimode microplate reader (Tecan, Switzerland), at 30 °C. The experiment was repeated using independent biological triplicates, and the averages of the measurements, with error bars representing standard deviations are shown.


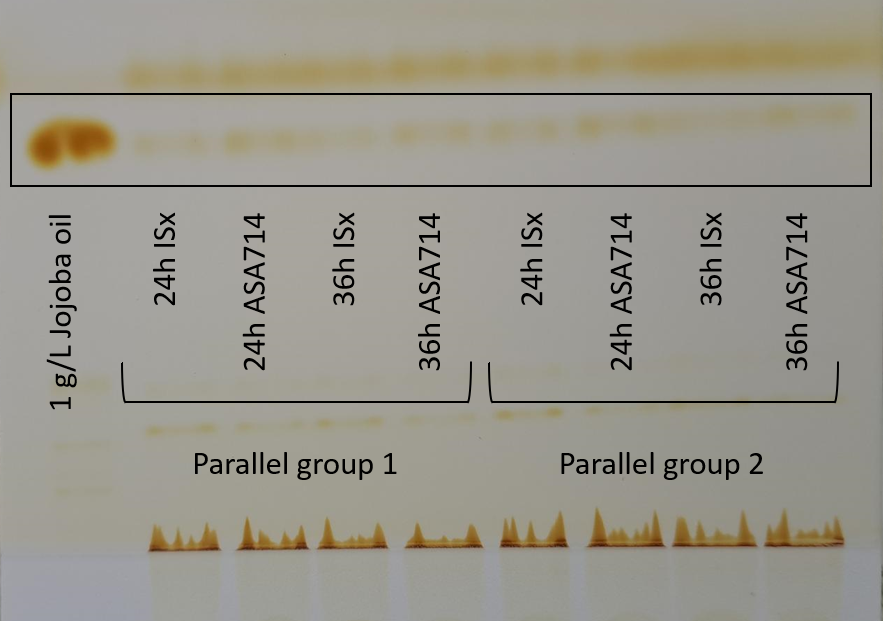


**Figure S4.** Visualization of WE accumulation of strains ADP1-ISx and ASA714 using thin layer chromatography (TLC). The cultivations were carried out at 30 °C, 300 rpm, using 100 mL Erlenmeyer flask with 10 mL MSM supplemented with 200 mM glucose, 300 rpm. The lipid extraction and the semi-quantitative analysis of WEs by TLC were done as described by Santala et al. [7]. Biomass samples were normalized to an OD_600_ of 20 of which 30 µL samples were applied on the TLC plate. TLC was carried out using Glass HPTLC Silica Gel 60F_254_ plates (Merck, USA) with a mobile phase of hexane: diethyl ether: acetic acid 90: 15: 1 (*v/v/v*). Wax esters on the TLC plates were visualized using iodine staining and using jojoba oil as the standard.


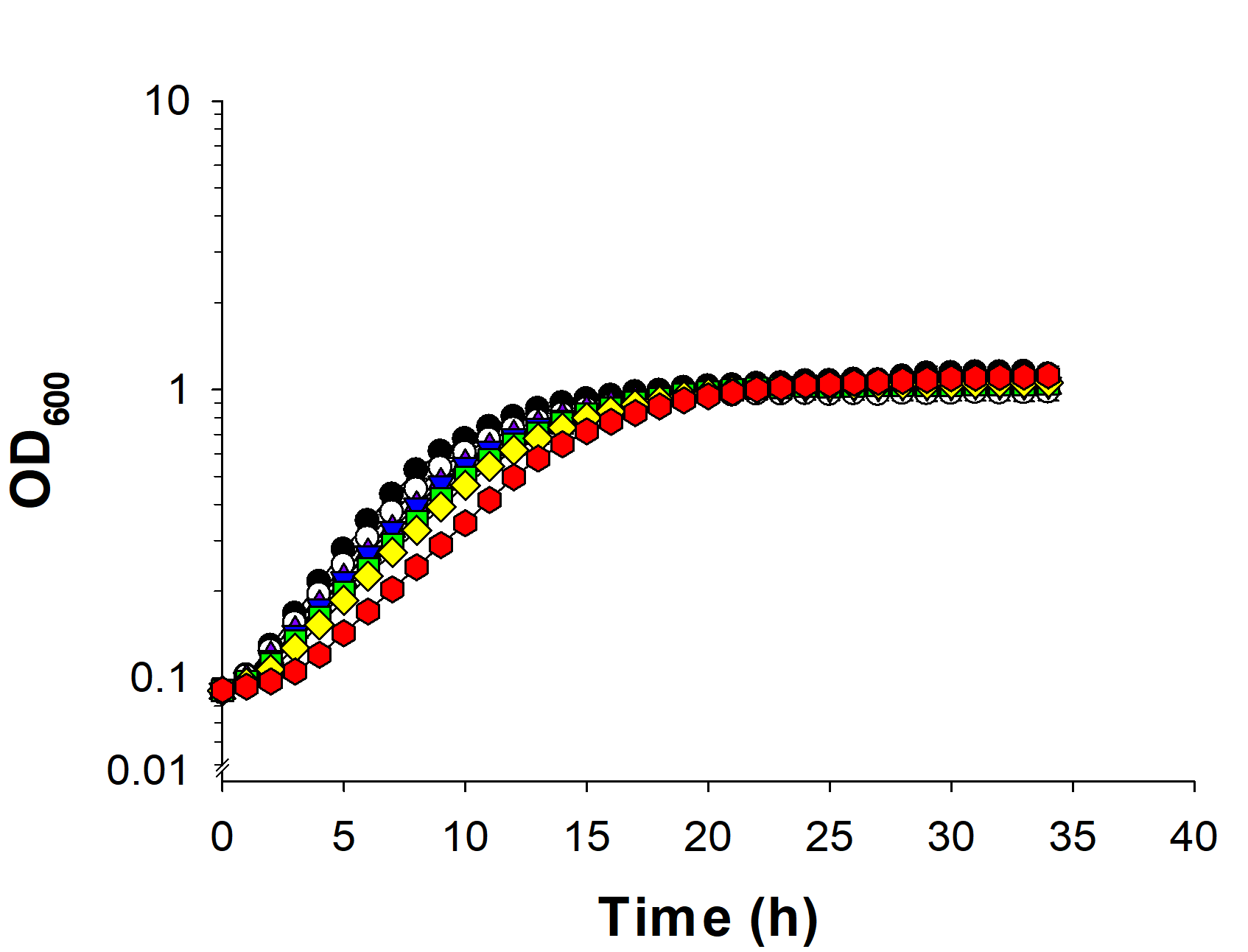


**Figure S5.** Growth of ASA714 in SLH medium at varying lactate concentrations. The experiment was repeated using independent biological triplicates. An overnight preculture was carried in 5 mL SLH medium at 30 °C, 300 rpm. The main culture was carried out using 96-well plate, 200 µL cultures with SLH medium supplemented with 0-18 g/L lactate, Spark multimode microplate reader (Tecan, Switzerland), at 30 °C. The averages of the measurements, with error bars representing standard deviations are shown. Error bars are present but may be obscured by data symbols.


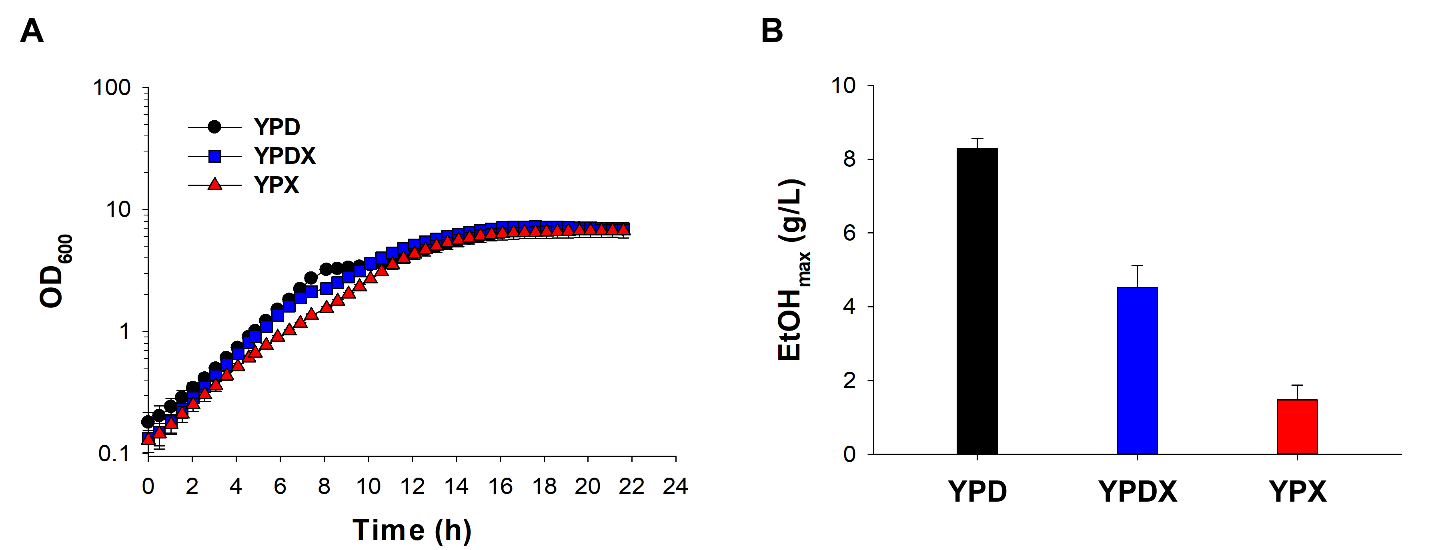


**Figure S6.** (A) Growth profiles of the XXX strain in YP media containing 20 g/L glucose, 10 g/L glucose and 10 g/L xylose or 20 g/L xylose for 24 h. (B) Maximum ethanol concentration in each media after 9 h fermentation. The experiment was repeated using independent biological triplicates, and the averages of the measurements, with error bars representing standard deviations are shown.


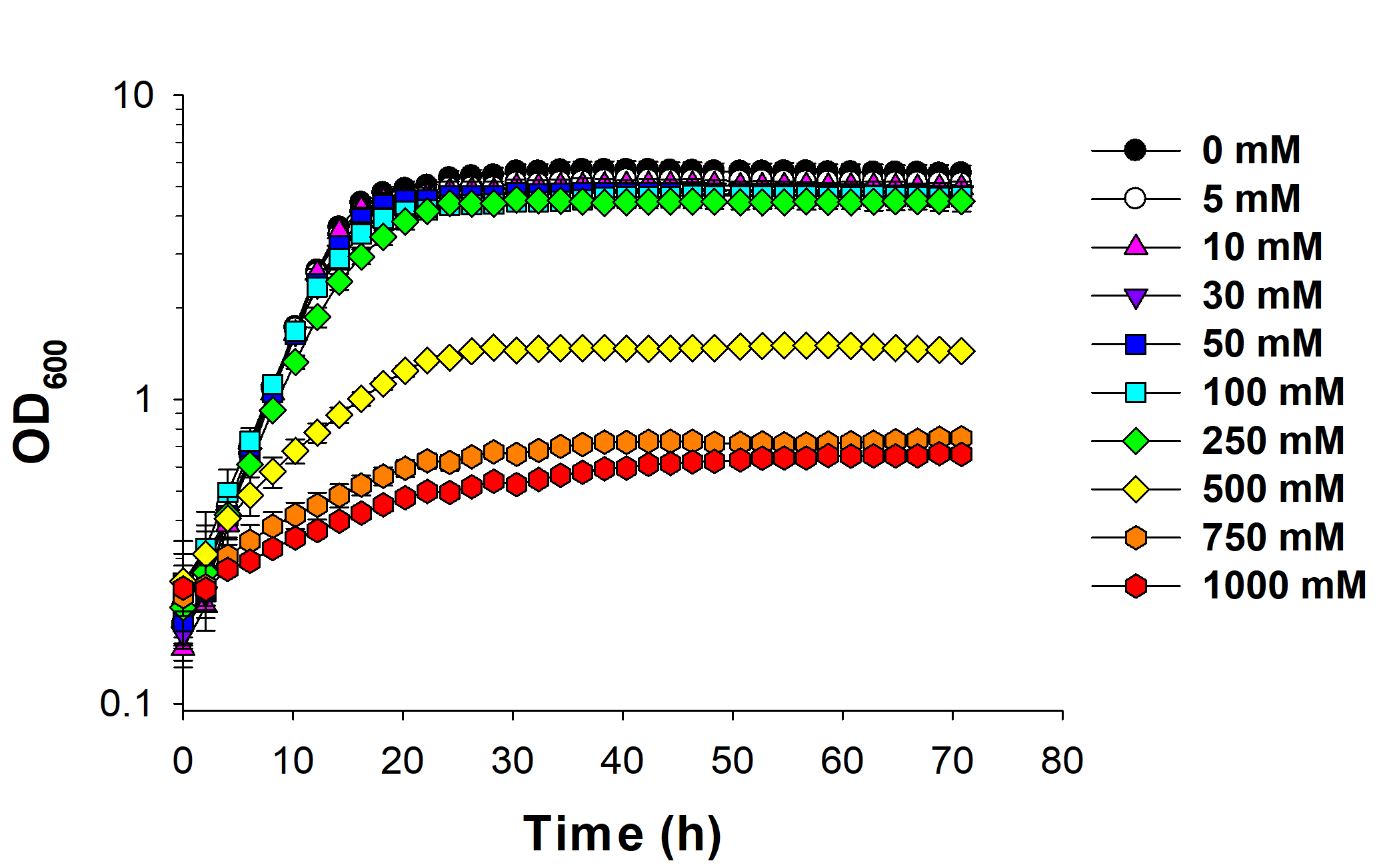


**Figure S7.** Growth profiles of the XXX strain in YPD medium supplemented with lactic acid at concentrations of 0, 5, 10, 30, 50, 100, 250, 500, 750, and 1000 mM lactic acid for 72 h. The experiment was repeated using independent biological triplicates, and the averages of the measurements, with error bars representing standard deviations are shown.

**
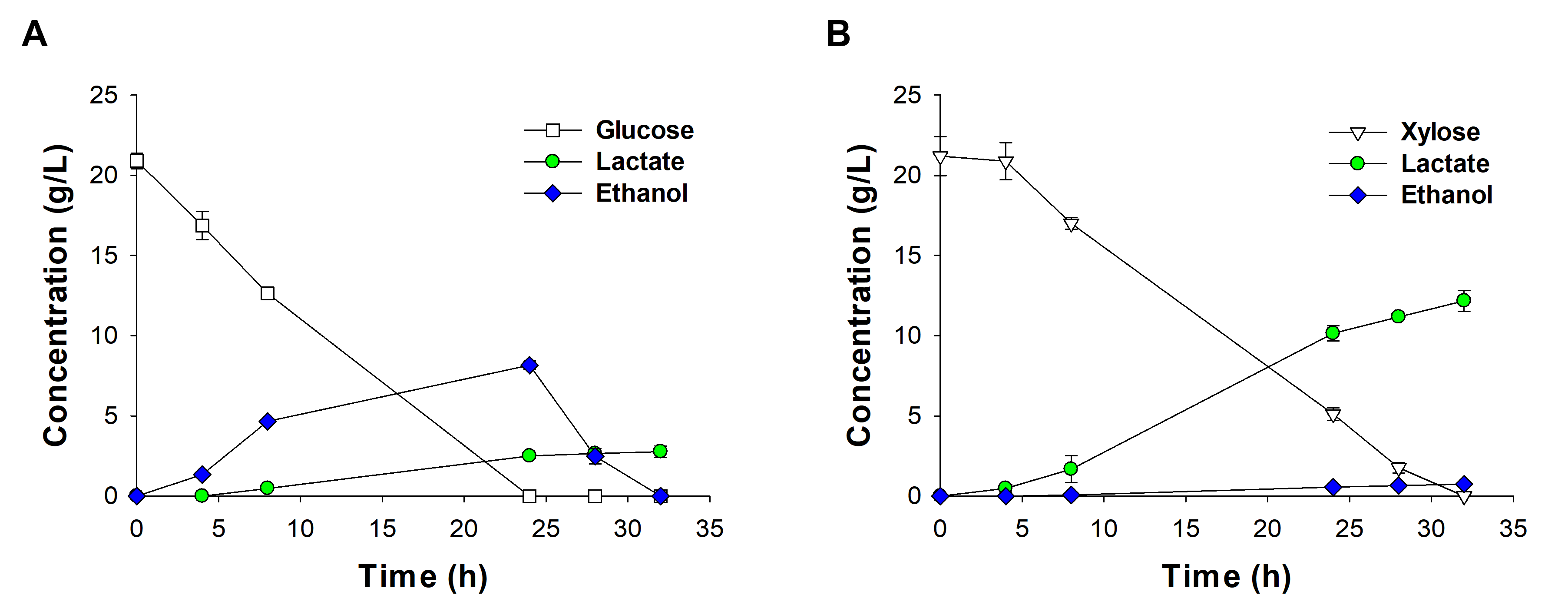
**

**Figure S8.** Fermentation kinetics of LX3 in (A) YPD or (B) YPX media for 32 h. The experiment was repeated using independent biological triplicates. The averages of the measurements, with error bars representing standard deviations are shown.


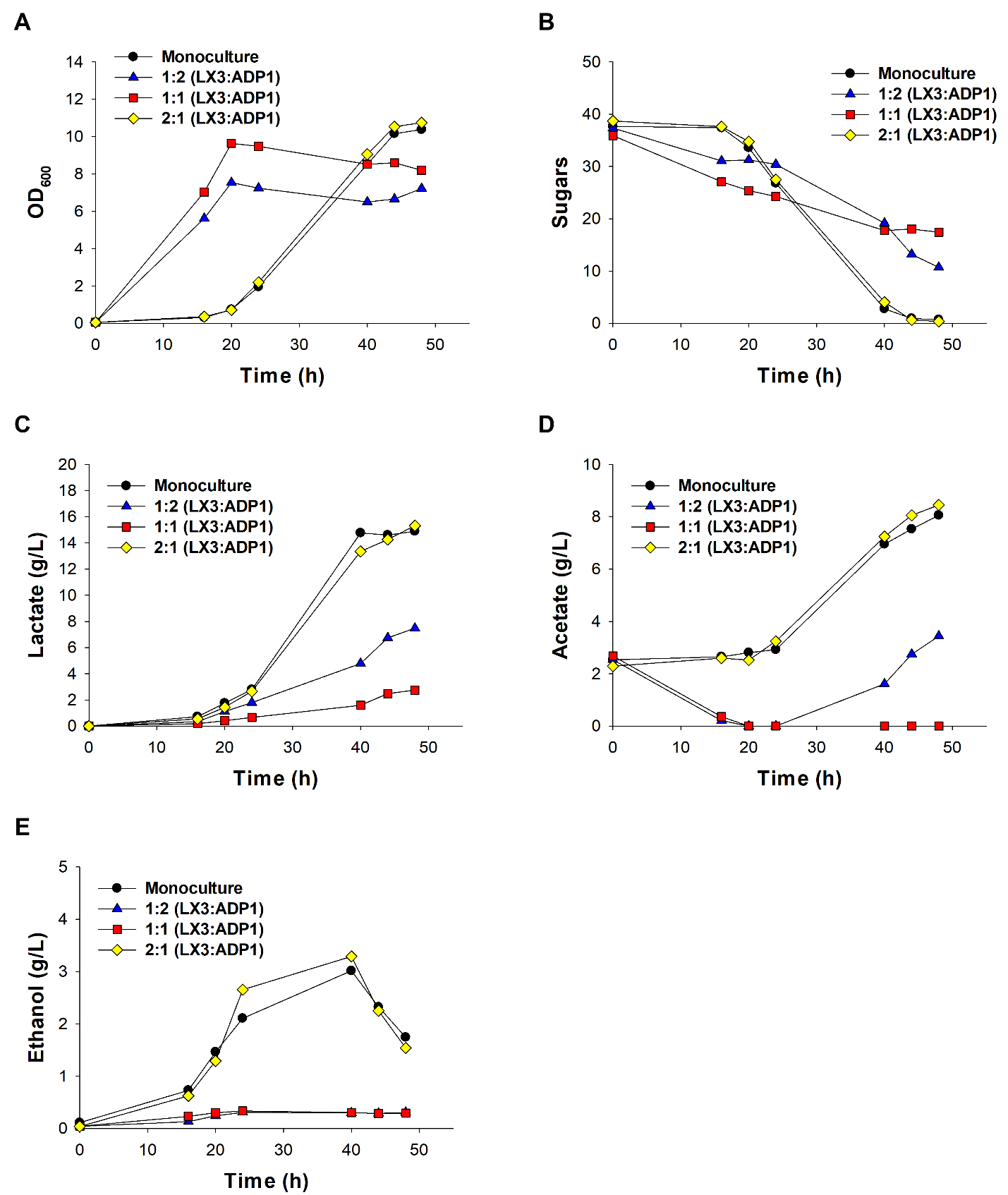


**Figure S9.** Fermentation kinetics including profiles of (A) cell growth, (B) sugar consumption, (C) lactate, (D) acetate, and (E) ethanol production of LX3 monoculture and coculture of LX3 and ADP1 at inoculation ratio of 1:2, 1:1, and 2:1. Fermentations were carried out in a 3.5 L bioreactor with 1 L SLH medium in 30 °C and pH was maintained at 6.0 by 2 M HCl and 8 % (*v/v*) NH_4_OH solutions.


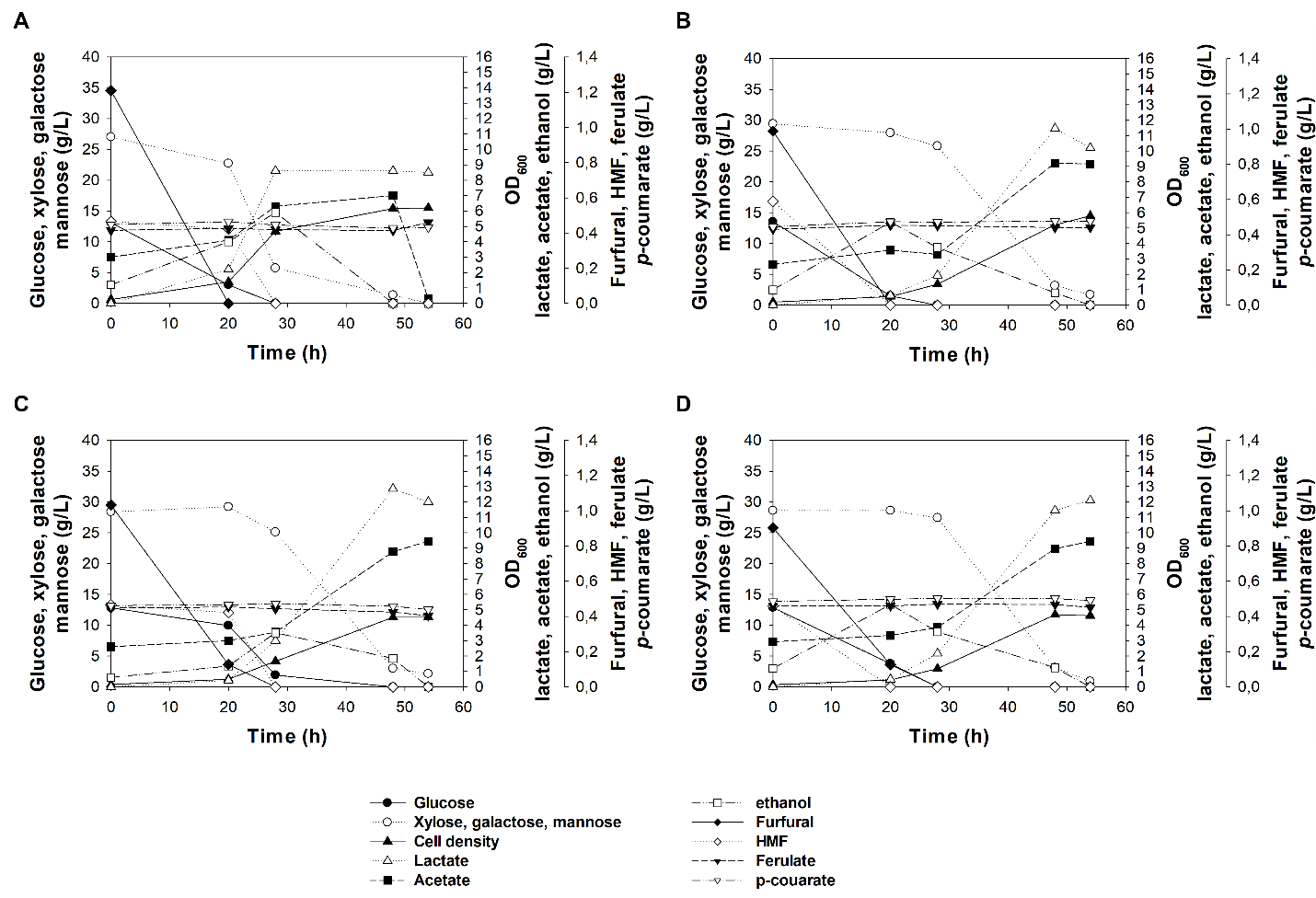


**Figure S10.** Fermentation kinetics of (A) 1^st^, (B) 2^nd^, (C) 3^rd^, and (D) 4^th^ LX3 monoculture in a bioreactor. Fermentations were carried out in a 3.5 L bioreactor with 1 L SLH medium at 30 °C and pH was maintained at 7.0 by 2 M HCl and 8 % (*v/v*) NH_4_OH solutions.


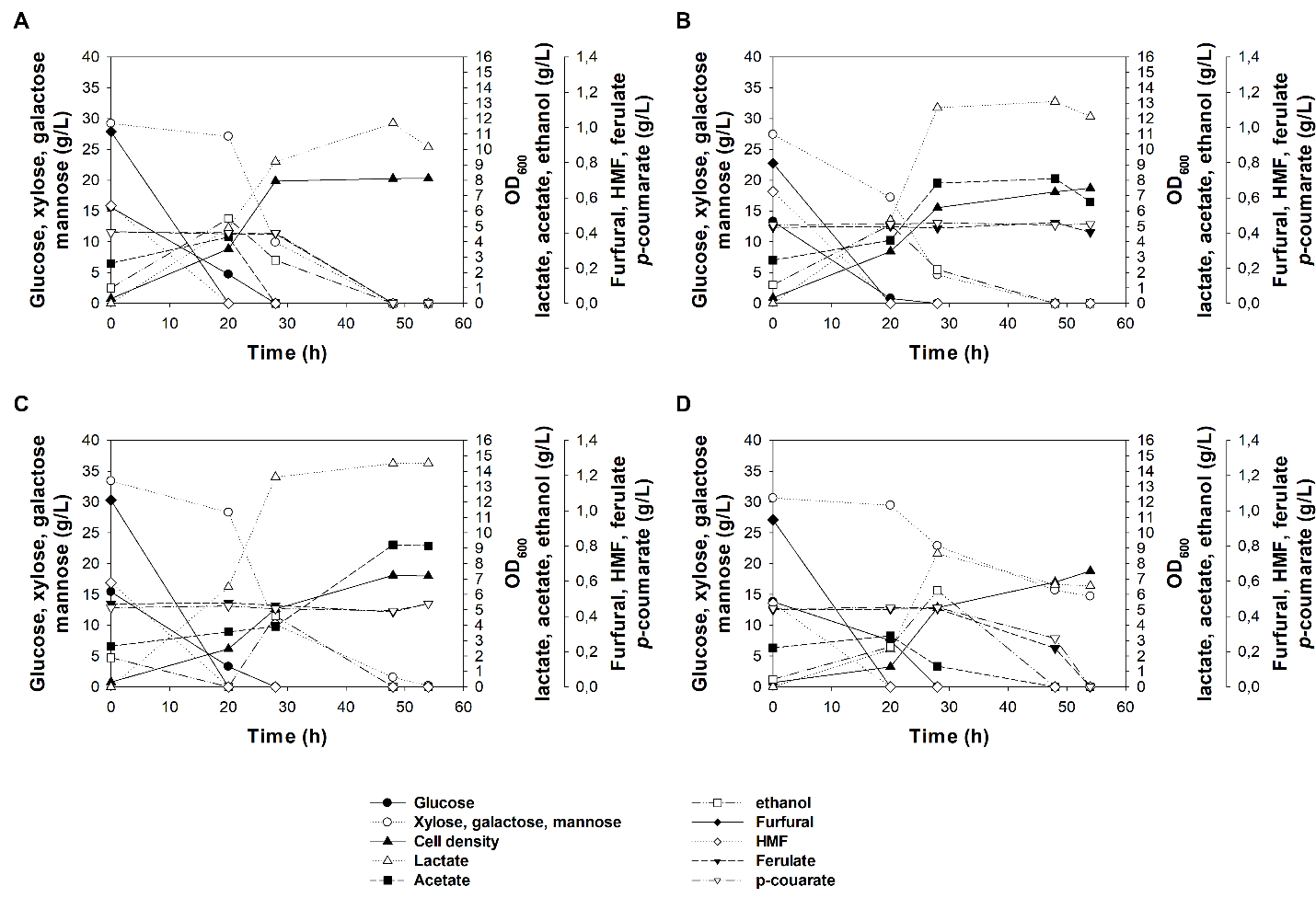


**Figure S11.** Fermentation kinetics of (A) 1^st^, (B) 2^nd^, (C) 3^rd^, and (D) 4^th^ LX3 and ASA714 coculture in a bioreactor. Fermentations were carried out in a 3.5 L bioreactor with 1 L SLH medium at 30 °C and pH was maintained at 7.0 by 2 M HCl and 8 % (*v/v*) NH_4_OH solutions.


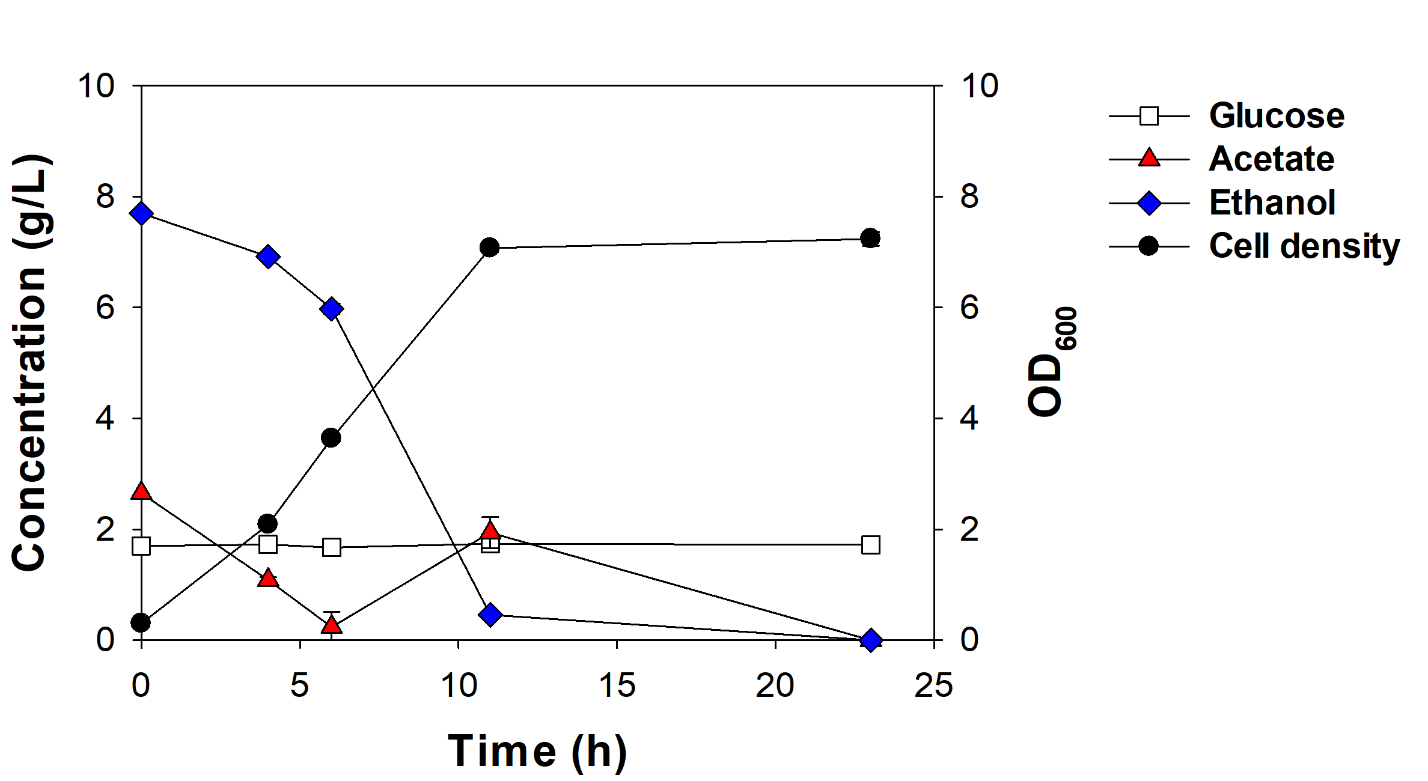


**Figure S12.** Growth and consumption of carbon sources by ASA714 in MSM supplemented with 8 g/L ethanol, 2.6 g/L acetate, and 2 g/L glucose. The cultivations were carried out using 100 mL Erlenmeyer flask with 10 mL cultivations, at 30 °C and 200 rpm. The experiment was repeated using independent biological replicates, and the averages of the measurements, with error bars representing standard deviations are shown.

**References**

1. de Berardinis V, Vallenet D, Castelli V, Besnard M, Pinet A, Cruaud C, et al. A complete collection of single-gene deletion mutants of Acinetobacter baylyi ADP1. Mol Syst Biol. 2008;4:174.

2. Luo J, Efimova E, Losoi P, Santala V, Santala S. Wax ester production in nitrogen-rich conditions by metabolically engineered Acinetobacter baylyi ADP1. Metab Eng Commun. 2020;10:e00128.

3. Luo J, Efimova E, Volke DC, Santala V, Santala S. Engineering cell morphology by CRISPR interference in Acinetobacter baylyi ADP1. Microb Biotechnol. 2022;15:2800–18.

4. Cámara E, Lenitz I, Nygård Y. A CRISPR activation and interference toolkit for industrial Saccharomyces cerevisiae strain KE6-12. Sci Rep. 2020;10:14605.

5. Losoi PS, Santala VP, Santala SM. Enhanced Population Control in a Synthetic Bacterial Consortium by Interconnected Carbon Cross-Feeding. ACS Synth Biol. 2019;8:2642–50.

6. Hartmans S, Smits JP, van der Werf MJ, Volkering F, de Bont JAM. Metabolism of Styrene Oxide and 2-Phenylethanol in the Styrene-Degrading Xanthobacter Strain 124X. Appl Environ Microbiol. 1989;55:2850–5.

7. Santala S, Efimova E, Kivinen V, Larjo A, Aho T, Karp M, et al. Improved Triacylglycerol Production in Acinetobacter baylyi ADP1 by Metabolic Engineering. Microbial Cell Factories. 2011;10:36.
